# Supplementary material for: The Prevotella copri Complex Comprises Four Distinct Clades Underrepresented in Westernized Populations
Source: Cell Host Microbe. 2019 Nov 13;26(5):666–679.e7. doi: 10.1016/j.chom.2019.08.018 (PMC6854460; doi:10.1016/j.chom.2019.08.018)
Supplement: Document S1. Figures S1–S5 [file mmc1.pdf]

Cell Host & Microbe, Volume *26*

Supplemental Information

The *Prevotella copri* Complex Comprises Four Distinct Clades Underrepresented in Westernized Populations

Adrian Tett, Kun D. Huang, Francesco Asnicar, Hannah Fehlner-Peach, Edoardo Pasolli, Nicolai Karcher, Federica Armanini, Paolo Manghi, Kevin Bonham, Moreno Zolfo, Francesca De Filippis, Cara Magnabosco, Richard Bonneau, John Lusingu, John Amuasi, Karl Reinhard, Thomas Rattei, Fredrik Boulund, Lars Engstrand, Albert Zink, Maria Carmen Collado, Dan R. Littman, Daniel Eibach, Danilo Ercolini, Omar Rota-Stabelli, Curtis Huttenhower, Frank Maixner, and Nicola Segata

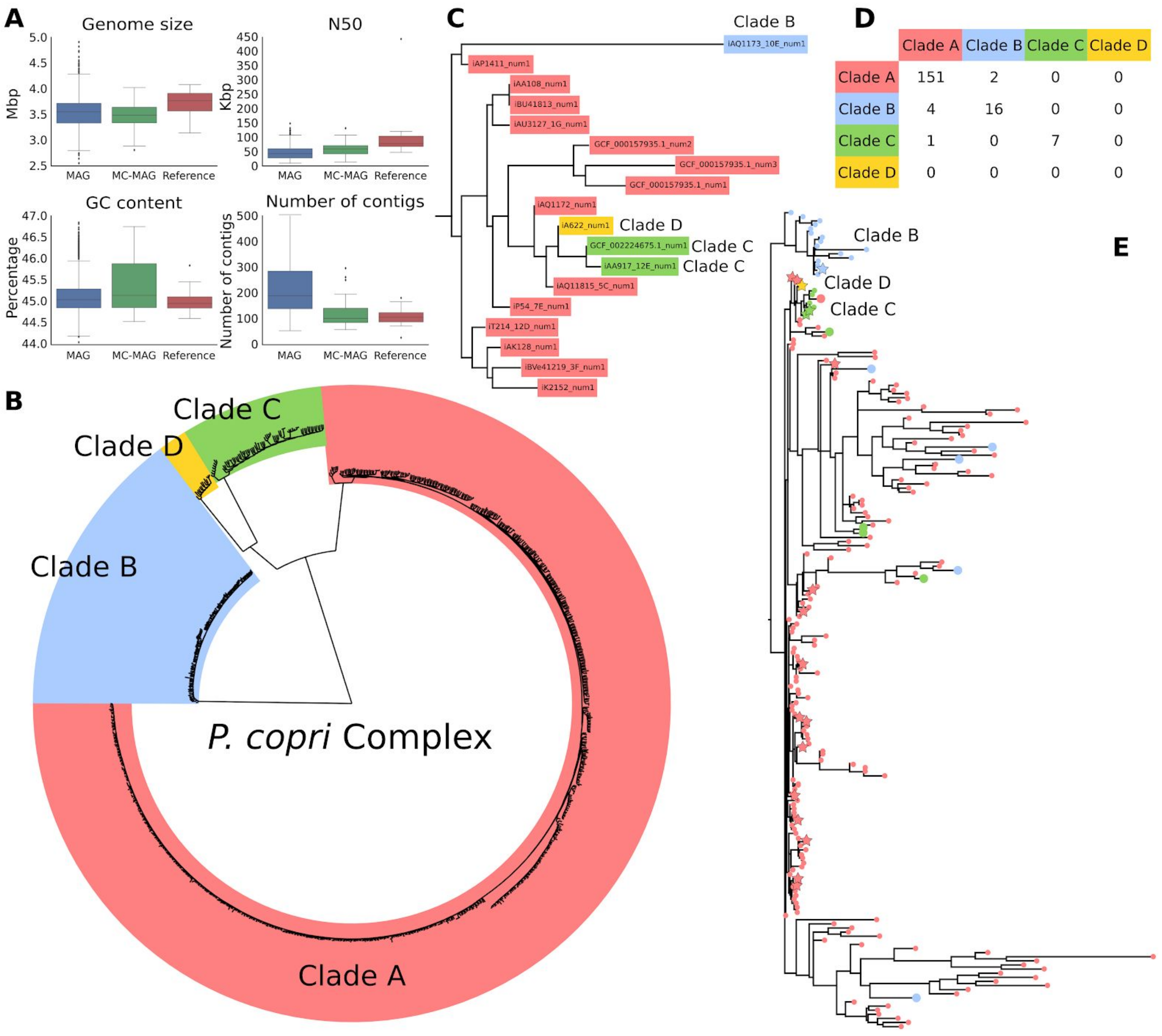

Supplementary Figure S1. ***P. copri*** **genome statistics and phylogenetic relatedness, related to Figure 1. A)** Comparison of genome statistics (Genome size, N50, GC% and number of contigs) for *P. copr*i isolate sequences (references = 17), for manually curated metagenome assembled genomes (MC-MAG = 55) and automatically metagenome assembled genomes (MAGs = 951). **B)** Phylogenetic representation of all 1023 *P. copri* genomes based on a set of 210 *P. copri* core genes (see **Methods**). **C-E**. Relatedness of the 16S rRNA gene sequences of the four *P. copri* clades reveals weak resolving power in discriminating the four clades. **C**, Phylogeny of all 16S rRNA gene sequences (>1000bp) recovered from all 17 isolate genomes using Barnap (https://github.com/tseemann/barrnap). **D**, Confusion matrix of all recovered 16S sequences (>1000bp) from all MAGs and their clade membership assigned based on the 16S rRNA gene sequence of the closest isolate genome (Blastn, >500bp alignment, identity > 85%) compared to their clade membership based on whole genome phylogenetic placement (panel **B**). **E**, 16S rRNA phylogenetic representation of all isolate genomes and MAGs. All alignments were produced using MAFFT (Katoh and Standley, 2013) and the following parameters: mafft --globalpair --maxiterate 1000 and visualised using FastTree with default parameters (Price et al., 2010).

A

# samples	Prevalence (Fisher) Any	Clade A	Clade B	Clade C	Clade D	Abundance (Mann-Whitney) Any	Clade A	Clade B	Clade C	Clade D	Dataset	Condition
66	27.3	21.2	7.6	12.1	3.0	6.7	4.1	0.4	7.6	0.6	ZellerG_2014	Control
42	35.7	33.3	11.9	9.5	4.8	11.6	8.8	1.5	4.2	13.5		Adenoma
91	26.4	20.9	11.0	7.7	4.4	6.6	5.4	2.4	0.9	6.4		CRC
133	29.3	24.8	11.3	8.3	4.5	8.6	6.8	2.1	2.1	8.8		Adenoma/CRC
61	8.2	3.3	1.6	3.3	1.6	1.0	0.5	1.5	0.8	1.1	FengQ_2015	Control
46	**39.1** *	**39.1** *	4.3	**17.4** *	0.0	**7.0** *	**6.4** *	0.9	**1.1** *	0.0		CRC
47	17.0	12.8	4.3	6.4	0.0	2.0	1.7	0.5	1.6	0.0		Adenoma
93	**28.0** *	**25.8** *	4.3	11.8	0.0	**5.5** *	**5.3** *	0.7	1.2	0.0		CRC/adenoma
52	23.1	23.1	1.9	0.0	0.0	11.7	11.7	0.2	0.0	0.0	VogtmannE_2016	Control
52	19.2	19.2	0.0	0.0	0.0	12.6	12.6	0.0	0.0	0.0		CRC
53	28.3	24.5	1.9	7.5	0.0	10.2	9.8	0.3	6.3	0.0	YuJ_2015	Control
75	30.7	28.0	5.3	4.0	0.0	10.0	10.2	2.2	2.7	0.0		CRC
174	29.9	25.9	4.0	8.0	1.7	28.6	26.4	10.2	15.1	5.2	QinJ_2012	Control
170	28.2	25.3	6.5	10.0	1.8	17.2	13.7	7.3	8.1	6.9		T2D
43	14.0	11.6	0.0	7.0	0.0	2.1	1.6	0.0	1.5	0.0	KarlssonFH_2013	Control
102	16.7	14.7	2.0	2.9	2.0	4.4	3.7	0.5	4.0	2.8		IGT/T2D
49	10.2	6.1	2.0	4.1	0.0	2.6	1.5	0.2	4.1	0.0		IGT
53	22.6	22.6	1.9	1.9	3.8	5.1	4.3	0.8	4.0	2.8		T2D
41	39.0	39.0	7.3	4.9	0.0	31.2	29.7	2.5	8.5	0.0	LiJ_2017	Control
99	48.5	47.5	11.1	16.2	2.0	37.3	31.6	4.2	13.8	18.6		Hypertension
56	53.6	48.2	3.6	**19.6** *	1.8	41.4	39.0	11.4	**14.9** *	2.1		Pre-hypertension
155	50.3	47.7	8.4	**17.4** *	1.9	38.9	34.3	5.3	**14.2** *	13.1		Pre-hypertension/hypertension
71	25.4	25.4	1.4	2.8	0.0	8.6	8.1	0.4	4.6	0.0	NielsenHB_2014	Control
148	33.8	29.7	**11.5** *	3.4	**6.1** *	11.5	10.1	**2.8** *	8.9	**4.5** *		IBD
53	26.4	24.5	1.9	11.3	3.8	15.1	12.5	1.1	6.7	3.4	HeQ_2017	Control
63	12.7	11.1	4.8	3.2	0.0	45.9	31.6	17.6	46.6	0.0		CD
114	52.6	48.2	1.8	13.2	1.8	9.6	9.1	4.8	3.9	2.1	QinN_2014	Control
123	58.5	57.7	2.4	16.3	0.8	11.7	9.7	1.5	6.8	14.5		Cirrhosis
36	11.1	8.3	5.6	2.8	0.0	25.4	26.2	10.0	3.0	0.0	RaymondF_2016	Control
36	19.4	13.9	11.1	5.6	0.0	16.0	17.6	4.6	2.8	0.0		Cephalosporins

B

	Clade A		Clade B		Clade C			
#Samples	Sub-1	Sub-2	Sub-1	Sub-2	Sub-1	Sub-2	Dataset	Condition
93	5.38	1.08	0.00	1.08	2.15	0.00	FengQ_2015	CRC/ADENOMA
61	0.00	0.00	0.00	1.64	0.00	0.00		Control
133	4.51	6.02	0.00	0.75	0.00	0.00	ZellerG_2014	CRC/ADENOMA
66	6.06	1.52	0.00	0.00	1.52	0.00		Control
52	3.85	13.46	0.00	0.00	0.00	0.00	VogtmannE_2016	CRC
52	0.00	21.15	0.00	0.00	0.00	0.00		Control
75	10.67	6.67	0.00	1.33	0.00	0.00	YuJ_2015	CRC
53	13.21	3.77	0.00	0.00	0.00	3.77		Control
170	10.59	2.94	0.00	1.76	2.35	0.00	QinJ_2012	T2D
174	14.94	2.87	0.00	1.72	1.72	0.57		Control
102	2.94	4.90	0.00	0.00	0.00	0.00	KarlssonFH_2013	IGT/T2D
43	2.33	0.00	0.00	0.00	0.00	2.33		Control
155	14.19	12.26	0.00	1.29	0.65	2.58	LiJ_2017	Pre-hypertension/hypertension
41	12.20	12.20	0.00	0.00	0.00	0.00		Control
148	6.76	7.43	0.00	0.68	0.00	0.00	NielsenHB_2014	IBD
71	8.45	8.45	0.00	0.00	0.00	0.00		Control
63	6.35	0.00	0.00	1.59	1.59	0.00	HeQ_2017	CD
53	9.43	0.00	0.00	0.00	0.00	3.77		Control
123	16.26	2.44	0.00	0.00	0.00	1.63	QinN_2014	Cirrhosis
114	11.40	3.51	0.00	1.75	0.88	0.88		Control
36	0.00	5.56	0.00	8.33	0.00	0.00	RaymondF_2016	Cephalosporins
36	0.00	2.78	0.00	2.78	0.00	0.00		Control

Supplementary Figure S2. The *P. copri* complex with respect to metagenomically investigated human diseases. Related to Figure 2. A, Prevalence and abundance of the *P. copri* complex in publicly available datasets for which there are case and control samples. * indicates p <0.05 (Fisher exact test (prevalence) or Mann-Whitney U test (abundance)) **B**, There is no significant association of *P. copri* sub-clades and disease (Fisher exact test), see **Methods** for inference of sub-clades.

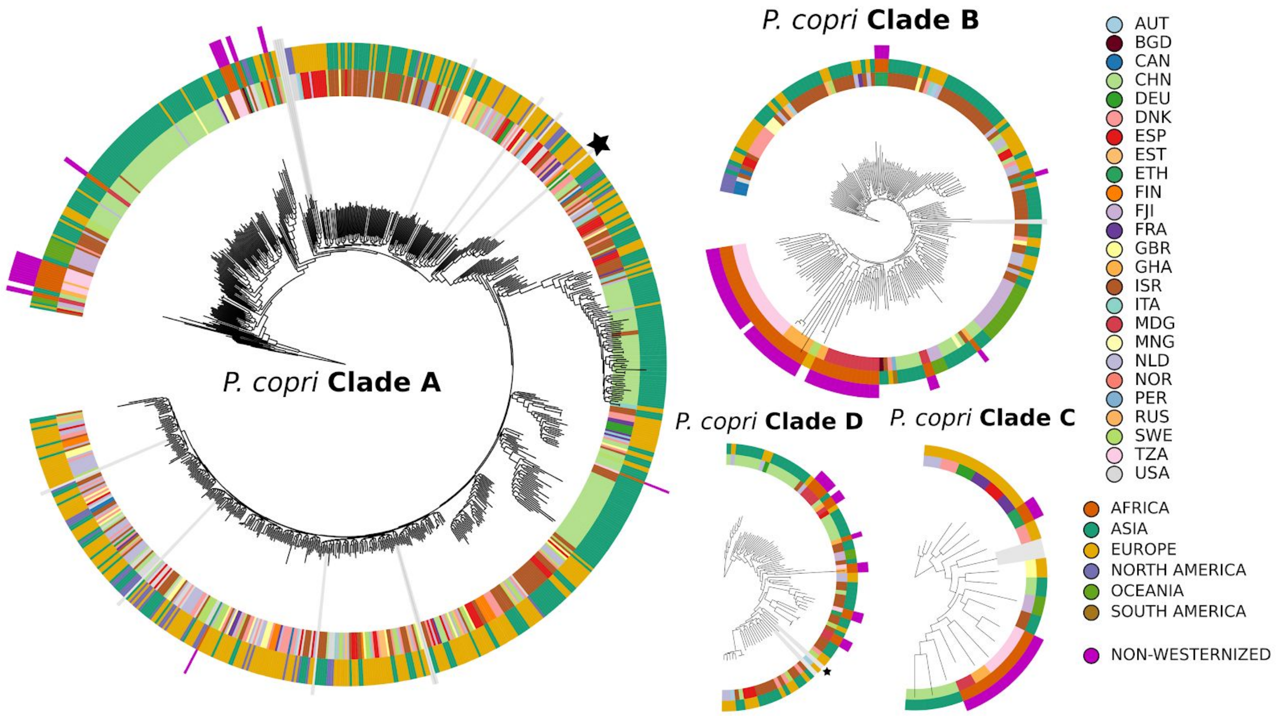

Supplementary Figure S3. Phylogeny of all 1121 *P. copri* genomes reconstructed in this study. Related to Figure 3. Outermost ring indicates if the genome was reconstructed from a non-Westernized sample (which includes the 98 genomes from our recently sequenced non-Westernized datasets), middle ring the continent and inner ring the country of origin. Publicly available *P. copri* references are indicated by black stars and our isolate genomes by radial gray bars.

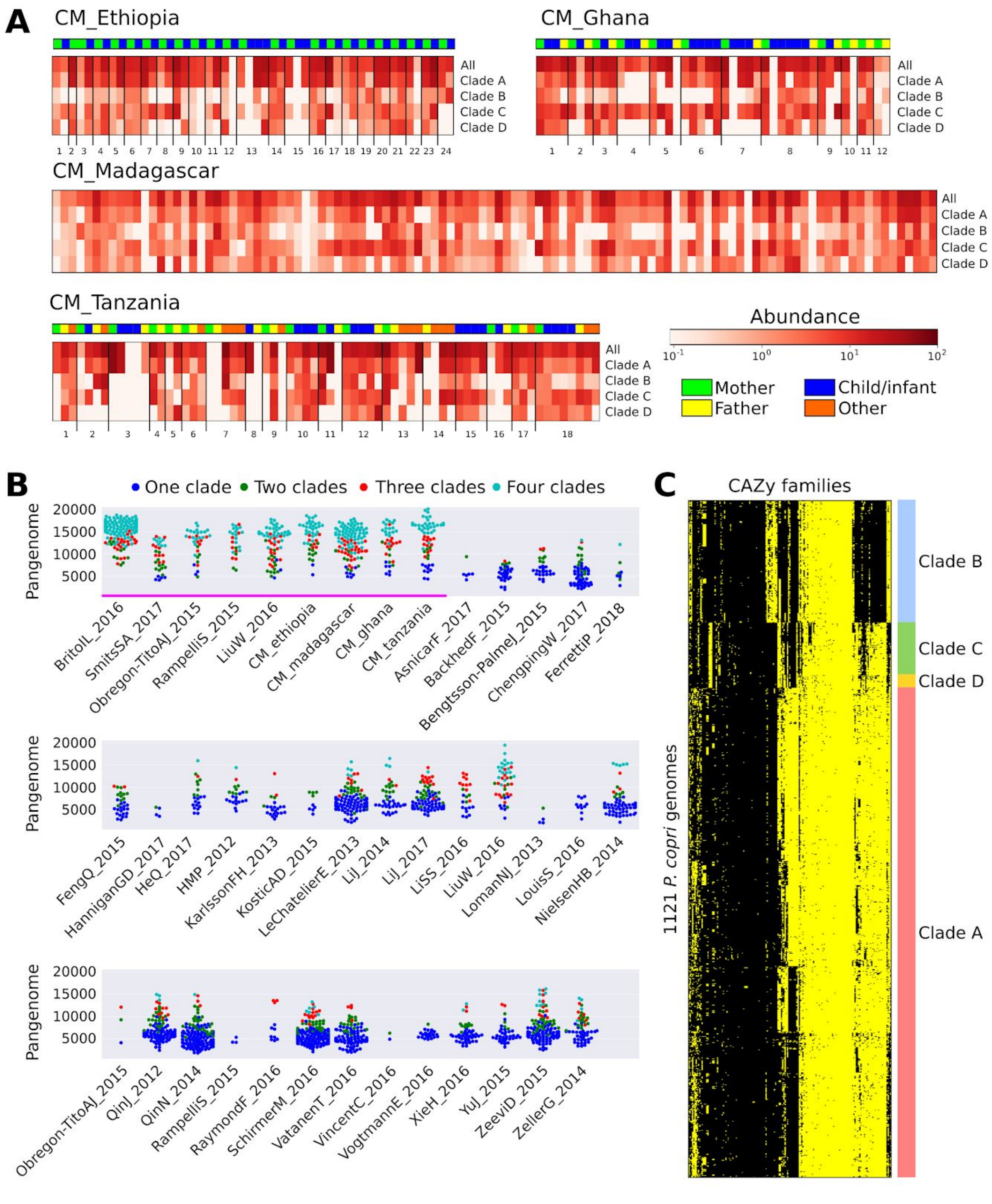

Supplementary Figure 4. **Abundance of *P. copri* in the recently sequenced non-Westernized datasets and functional diversity of the *P. copri* complex. Related to Figures 3 and 4. A**, Co-presence and abundance of the *P. copri* complex in our recently sequenced Non-westernized datasets. For datasets from Ethiopia, Ghana and Tanzania numbers refers to family membership. **B**, The within sample *P. copri* complex pangenome for datasets considered in this study. Non-Westernized datasets are underlined in magenta. **C**, Presence/absence heatmap of CAZy families in each of the 1121 *P. copri* genomes (yellow present, black absent)

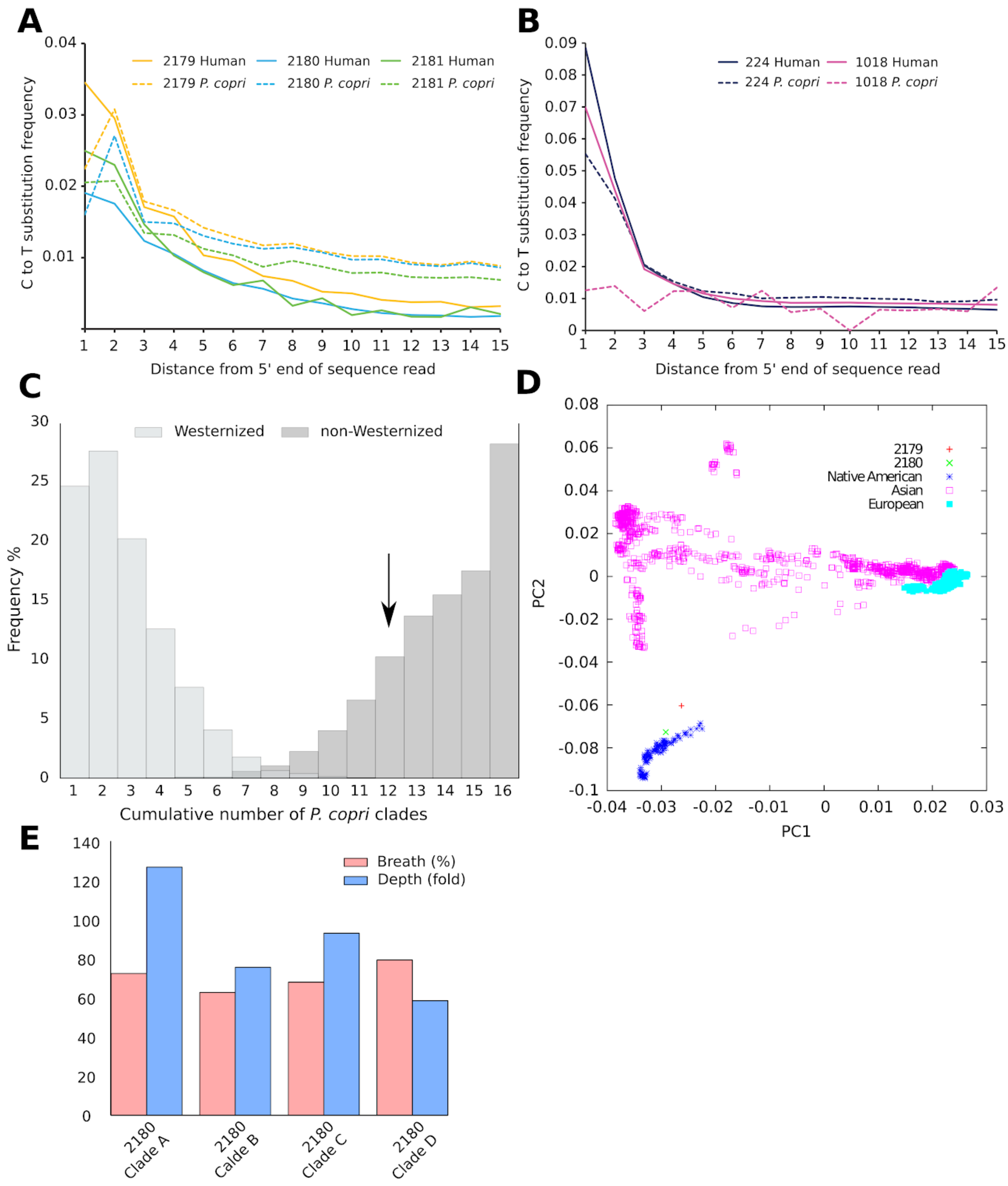

Supplementary Figure S5. **Analysis of the ancient ice-mummy and pre-Columbian amerind metagenomic samples. Related to Figure 5 and STAR methods.** Ancient DNA damage profiles. Cytosine to thymine substitution frequencies in the 5´ end of the human and *P. copri* (dashed lines) sequence reads detected in the Mexican coprolite material **(A)** and in the Iceman samples **(B)**. **C**, Co-presence of *P. copri* clades in ancient individuals is similar to contemporary non-Westernized individuals. Random subsampling of four individuals from either non-Westernized or Westernized populations and the cumulative number of *P. copri* clades observed (min 0, max 16). Subsampling repeated 10,000 times for each population. Black arrow indicates the number observed in the four ancient samples. **D**, PCA plot of two Mexican coprolite samples and selected modern European, Asian and Native American. Genome-wide ancient data was projected against a selected subset of the Affymetrix Human Origins populations. **E**, Depth and breadth of metagenomic reads from

sample 2180 mapped against four *P. copri* isolate genomes representing the four clades in the *P. copri* complex.
