# Supplementary material for: The Prevotella copri Complex Comprises Four Distinct Clades Underrepresented in Westernized Populations
Source: Cell Host Microbe. 2019 Nov 13;26(5):666–679.e7. doi: 10.1016/j.chom.2019.08.018 (PMC6854460; doi:10.1016/j.chom.2019.08.018)
Supplement: Document S2. Article plus Supplemental Information [file mmc7.pdf]

Article

Cell Host & Microbe

The *Prevotella copri* Complex Comprises Four Distinct Clades Underrepresented in Westernized Populations

Graphical Abstract

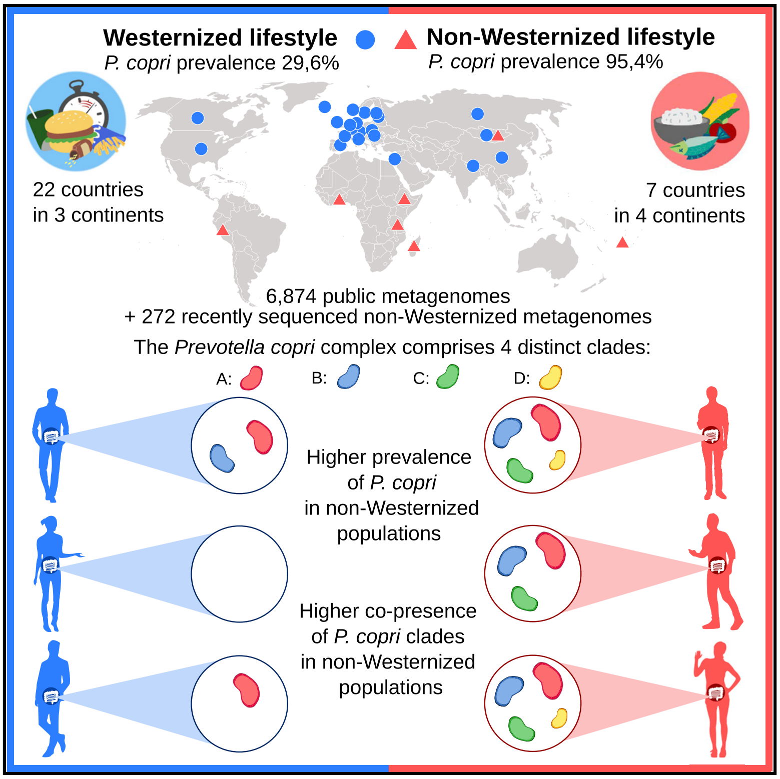

Highlights

- *P. copri* is not a monotypic species but composed of four distinct clades
- The *P. copri* complex is more prevalent in populations with non-Westernized lifestyles
- *P. copri* clades are frequently co-present within non-Westernized individuals
- Ancient stool samples suggest Westernization leads to *P. copri* underrepresentation

Authors

Adrian Tett, Kun D. Huang, Francesco Asnicar, ..., Curtis Huttenhower, Frank Maixner, Nicola Segata

Correspondence

adrianjames.tett@unitn.it (A.T.), nicola.segata@unitn.it (N.S.)

In Brief

Tett et al. find that the intestinal microbe *Prevotella copri* encompasses four distinct clades constituting the *P. copri* complex. The complex is prevalent in non-Westernized populations where co-presence of all clades is commonly observed within individuals. Analysis of ancient stool samples supports Westernization as contributing to reduced *P. copri* prevalence.

Tett et al., 2019, Cell Host & Microbe *26*, 666–679
November 13, 2019 © 2019 The Author(s). Published by Elsevier Inc.
https://doi.org/10.1016/j.chom.2019.08.018

CellPress

Cell Host & Microbe
Article

The *Prevotella copri* Complex Comprises Four Distinct Clades Underrepresented in Westernized Populations

Adrian Tett,[1,*] Kun D. Huang,[1,2] Francesco Asnicar,[1] Hannah Fehlner-Peach,[3] Edoardo Pasolli,[1,19] Nicolai Karcher,[1] Federica Armanini,[1] Paolo Manghi,[1] Kevin Bonham,[4,6] Moreno Zolfo,[1] Francesca De Filippis,[6,7] Cara Magnabosco,[8] Richard Bonneau,[8,9] John Lusingu,[10] John Amuasi,[11] Karl Reinhard,[12] Thomas Rattei,[13] Fredrik Boulund,[14] Lars Engstrand,[15] Albert Zink,[15] Maria Carmen Collado,[16] Dan R. Littman,[3] Daniel Eibach,[17,18] Danilo Ercolini,[6,7] Omar Rota-Stabelli,[2] Curtis Huttenhower,[4,5] Frank Maixner,[15] and Nicola Segata[1,20,*]
[1]CIBIO Department, University of Trento, 38123 Trento, Italy
[2]Department of Sustainable Agro-Ecosystems and Bioresources, Fondazione Edmund Mach, 1 38010 S, San Michele all'Adige, Italy
[3]Kimmel Center for Biology and Medicine of the Skirball Institute, New York University School of Medicine, New York, NY 10016, USA
[4]The Broad Institute of MIT and Harvard, Cambridge, MA 02115, USA
[5]Biostatistics Department, Harvard T.H. Chan School of Public Health, Boston, MA 02115, USA
[6]Department of Agricultural Sciences, University of Naples "Federico II", Portici, Italy
[7]Task Force on Microbiome Studies, University of Naples "Federico II", Naples, Italy
[8]Center for Computational Biology, Flatiron Institute, New York, NY 10010, USA
[9]Departments of Biology and Computer Science, New York University, New York, NY 10003, USA
[10]National Institute for Medical Research, Tanga Centre, Tanzania
[11]Kumasi Centre for Collaborative Research in Tropical Medicine, Kwame Nkrumah University of Science and Technology, Ghana
[12]Hardin Hall, School of Natural Resources, University of Nebraska, Lincoln, NE 68583-0987, USA
[13]CUBE - Division of Computational Systems Biology, Department of Microbiology and Ecosystem Science, University of Vienna, Althanstrasse 14, 1090 Vienna, Austria
[14]Centre for Translational Microbiome Research, Department of Microbiology Tumor and Cell Biology, Karolinska Institutet, 171 65 Solna, Stockholm, Sweden
[15]Institute for Mummy Studies, EURAC Research, Viale Druso 1, 39100 Bolzano, Italy
[16]Institute of Agrochemistry and Food Technology, National Research Council (IATA-CSIC), 46980 Paterna, Valencia, Spain
[17]Department of Infectious Disease Epidemiology, Bernhard Nocht Institute for Tropical Medicine, 20359 Hamburg, Germany
[18]German Center for Infection Research, Hamburg-Borstel-Lübeck-Riems, 20359 Hamburg, Germany
[19]Present address: Department of Agricultural Sciences, University of Naples "Federico II," Portici, Italy
[20]Lead Contact
*Correspondence: adrianjames.tett@unitn.it (A.T.), nicola.segata@unitn.it (N.S.)
https://doi.org/10.1016/j.chom.2019.08.018

SUMMARY

Prevotella copri **is a common human gut microbe that has been both positively and negatively associated with host health. In a cross-continent meta-analysis exploiting >6,500 metagenomes, we obtained >1,000 genomes and explored the genetic and population structure of *P. copri*. *P. copri* encompasses four distinct clades (>10% inter-clade genetic divergence) that we propose constitute the *P. copri* complex, and all clades were confirmed by isolate sequencing. These clades are nearly ubiquitous and co-present in non-Westernized populations. Genomic analysis showed substantial functional diversity in the complex with notable differences in carbohydrate metabolism, suggesting that multi-generational dietary modifications may be driving reduced prevalence in Westernized populations. Analysis of ancient metagenomes highlighted patterns of *P. copri* presence consistent with modern non-Westernized populations and a clade delineation time pre-dating human migratory waves out of Africa. These findings reveal that *P. copri* exhibits a high diversity that is underrepresented in Western-lifestyle populations.**

INTRODUCTION

Prevotella copri is a frequent inhabitant of the human intestinal microbiome, and it displays a large inter-individual variation (Human Microbiome Project Consortium, 2012; Qin et al., 2010; Truong et al., 2017). *P. copri* is 39.1% prevalent in healthy individuals from current metagenomic profiles (Pasolli et al., 2017); as such, it is not ubiquitous, but when present, it is often the most abundant species identified (34% of instances).

Interest in *P. copri* has gathered pace in part due to its reported association with inflammatory diseases (Dillon et al., 2014; Scher et al., 2013; Wen et al., 2017) and insulin resistance and glucose intolerance (Pedersen et al., 2016). Conversely, others have linked *P. copri* with improved glucose and insulin tolerance in diets rich in fiber (De Vadder et al., 2016; Kovatcheva-Datchary et al., 2015), which suggests the beneficial effects of *P. copri* could be diet dependent (Pedersen et al., 2016). As previously expressed (Cani, 2018; Ley, 2016), such conflicting

 © 2019 The Author(s). Published by Elsevier Inc.
This is an open access article under the CC BY license (http://creativecommons.org/licenses/by/4.0/).

reports regarding the benefits of *P. copri* suggest that it is an important but enigmatic member of the gut microbiome.

Higher prevalence of *Prevotella* has been consistently reported in non-Westernized populations (De Filippo et al., 2010; Hansen et al., 2019; Obregon-Tito et al., 2015; Schnorr et al., 2014; Smits et al., 2017; Yatsunenko et al., 2012), and metagenomic studies, capable of species-level resolution, have shown *P. copri* to be particularly prevalent (Pasolli et al., 2019; Vangay et al., 2018). Non-Westernized populations follow a traditional lifestyle and typically consume diets rich in fresh unprocessed food (vegetables and fruits). Although Westernization encompasses more factors and lifestyle modifications than diet alone, as discussed previously (Brewster et al., 2019; Pasolli et al., 2019), the association of *Prevotella* and Westernization may further support the hypothesis of diet being an important factor in selecting and shaping *Prevotella* populations. Indeed, diet has previously been shown to be important in the overall diversity of intestinal microbial communities (Smits et al., 2017; Sonnenburg and Bäckhed, 2016; Sonnenburg et al., 2016). The higher prevalence of *Prevotella* in societies following a more traditional healthy diet than the typical Westernized diet may also lend support for the health benefit of *P. copri*.

Despite the importance of *P. copri* and the open-ended question regarding its role in health and disease, there is a lack of available reference genomes, and much of our understanding of *P. copri* has been gathered from studies relying on the type strain *P. copri* DSM-18205 (Hayashi et al., 2007). Recent reports have begun to highlight a degree of strain-level heterogeneity within *P. copri* (De Filippis et al., 2019; Truong et al., 2017; Vangay et al., 2018). Indeed, sub-species strain variation may account for at least some of the differences in the reported benefits or detriments of *P. copri*. Yet, to date, there has been no large-scale concerted effort to explore the distribution and genetic variation within *P. copri*.

Here, we use a combination of isolate sequencing and large-scale metagenomic assembly and strict quality control to reconstruct over 1,000 *P. copri* genomes from publicly available metagenomes spanning multiple countries, diseases, and lifestyles. We also expand the catalog of non-Westernized sampled populations with additional metagenomic sequencing of individuals from Ghana, Ethiopia, and Tanzania and further profile *P. copri* in ancient intestinal samples from a European natural ice mummy and stools of pre-Columbian Amerinds. These datasets and analyses provide an unprecedented comprehensive insight into the genetic diversity, global population structure, and evolutionary history of *P. copri*.

RESULTS

Analysis of >1,000 *P. copri* Genomes Reveals Four Clades Comprising the *P. copri* Complex

To investigate the global distribution and population structure of *P. copri*, we performed an analysis of 6,874 publicly available metagenomes from 36 individual datasets (Table S1), representing six continents and 25 different countries. By means of an assembly and mapping-based computational approach, we expanded the total number of available *P. copri* genomes to 1,023, and the metagenome assembled genomes included in this set can be defined as high quality according to current guidelines (Bowers et al., 2017) (estimated completeness >95% and contamination <5%, see STAR Methods). This approach (see STAR Methods) involved collating a highly representative set of genomes comprising our recently sequenced *P. copri* isolates (n = 15), publicly available reference isolates (n = 2), as well as a set of carefully curated and manually guided metagenome assembled genomes from diverse populations (n = 55). This set of 72 genomes was used as a pangenomic reference to bin via mapping metagenomically assembled contigs from single samples into whole *P. copri* genomes (n = 951) (see STAR Methods). Therefore, of the 1,023 genomes, 17 are sequence isolates and 1,006 are metagenome-assembled genomes (MAGs). All the 1,006 MAGs passed strict quality control including estimation of within sample strain heterogeneity (see STAR Methods) and resulted in genomes with assembly characteristics comparable with those of isolate sequencing (Figure S1A; Table S1). This genome catalog spans multiple host geographies, populations, and lifestyles that can be mined to answer fundamental questions regarding the genomic structure of *P. copri*.

Strikingly, this analysis revealed that *P. copri* is not a monotypic species but is composed of four distinct clades when placed in phylogenetic context with the closest publicly available representatives of the wider *Prevotella*, *Alloprevotella*, and *Paraprevotella* genera (Figure 1A). These four clades are clearly distinct to other *Prevotella* species and to the other considered species; each clade is supported by at least one of our recently sequenced isolate genomes (Figure 1A), and all clades are represented in our recently sequenced non-Westernized datasets (98 additional genomes, see below). The average nucleotide identity (ANI) distances between the *P. copri* genomes revealed a limited intra-clade distance (mean 2.55% SD 0.35% for clade B to 4.16% SD 0.78% for clade C). Conversely, the inter-clade distances were very high, with values ranging from 13.0% to 21.4%. In comparison, each of the four *P. copri* clades were >23.0% distant to any other *Prevotella*, *Alloprevotella*, and *Paraprevotella* species, indicating that the four distinct *P. copri* clades are genetically closer to each other than genomes outside the four *P. copri* clades (Figure 1B).

The high inter-clade genetic distance observed suggests the genomes could represent four distinct species. Studies have sought to place a threshold at which ANI values between genomes equate to the delineation of strains into species, with a broad consensus being values above 5%–6% distance (Goris et al., 2007; Jain et al., 2018; Konstantinidis and Tiedje, 2005; Pasolli et al., 2019). All members of all four clades fall well below this threshold when compared to other *P. copri* clades (>10% ANI distance) (Figure 1B). The distinction of the four clades is further supported based on core genome single nucleotide distance (>10% distance) (Figure 1C), by the separation of the clades based purely on gene content (Figure 1D) as well as based on phylogeny (Figure 1A). Nevertheless, analysis of the 16S rRNA gene alone is insufficient to distinguish these clades (Figures S1C–S1E), which is not uncommon for species within the same genus (Janda and Abbott, 2007). Respecting the clear distinction of the *P. copri* clades and being conscious of the difficulties in advising separation into species, we propose the naming of *P. copri* to encompass these four distinct clades. Therefore, we propose the term ''*Prevotella copri* complex'' for which there

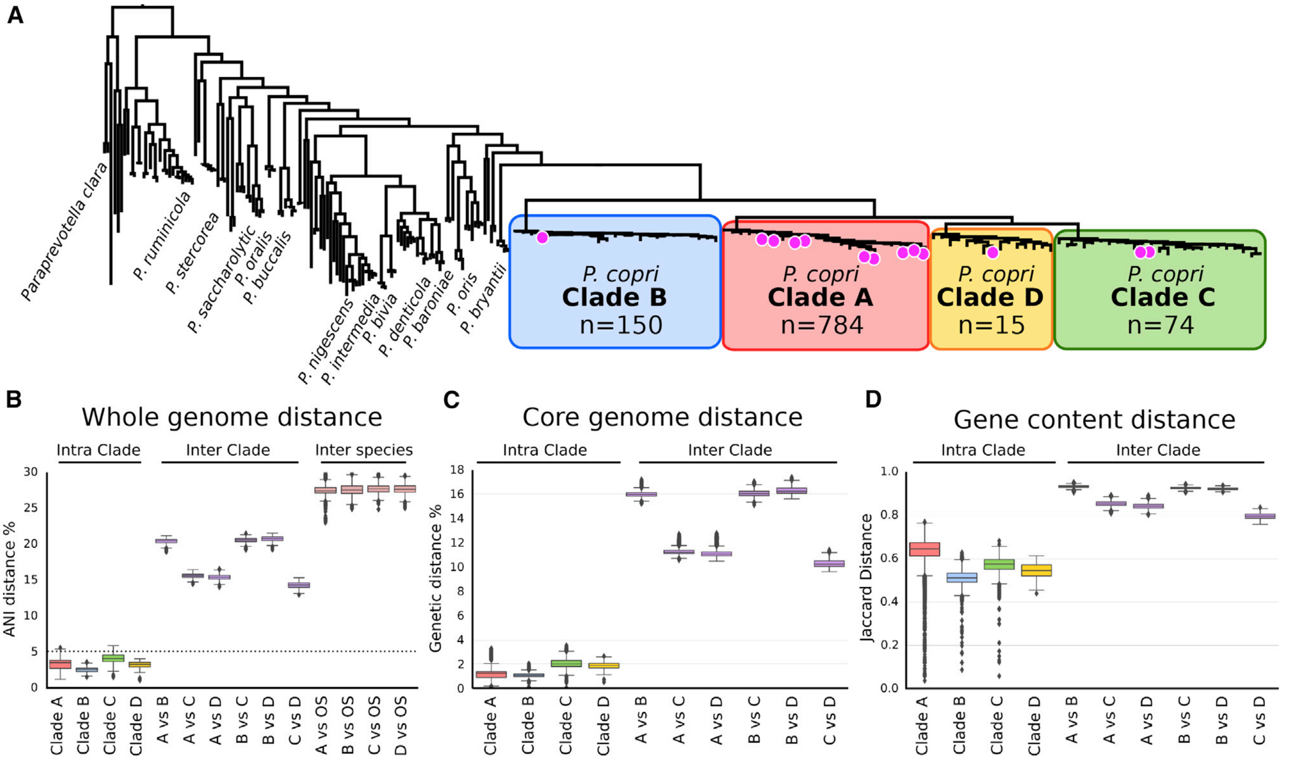

Figure 1. The Four Distinct Clades of the *P. copri* Complex

(A) Whole-genome phylogenetic tree of a representative subset of the four *P. copri* clades comprising the *P. copri* complex in relation to other sequenced members of the genera *Prevotella*, *Alloprevotella*, and *Paraprevotella*. Magenta circles indicate *P. copri* isolate sequences (built using 400 universal bacterial marker gene sequences, see STAR Methods). The phylogeny containing all *P. copri* genomes is available as Figure S1B and http://segatalab.cibio.unitn.it/data/Pcopri_Tett_et_al.html (see Data and Code Availability; Method Details).

(B) Genetic distances within a clade (intra-clade), between clades (inter-clade), and between clades and other species (denoted as OS) of *Prevotella*, *Alloprevotella*, and *Paraprevotella* (inter-species), shown as pairwise average nucleotide identity distances (ANI distance). The dotted line denotes 5% ANI distance.

(C) Pairwise SNV distances based on core gene alignment within and between clades (see STAR methods).

(D) Jaccard distance based on pairwise gene content (see STAR Methods) between and within the *P. copri* clades.

are four genetically distinct clades (A, B, C, and D), named sequentially based on the decreasing number of genomes reconstructed (Figure 1A).

The Four Clades Are Globally Distributed with Instances of Country-Specific Sub-types

In this study, *P. copri* genomes were reconstructed from 22 different countries offering a unique opportunity to investigate the biogeographical population structure. These clades were not strictly separated based on geographical location, i.e., all of the four *P. copri* clades were identified in multiple countries and spanning multiple continents (Figure 2). However, within several clades, we did observe geographical stratification. In clade A, for which the most genomes were reconstructed, we observed three sub-types that were either exclusive or nearly so to samples of Chinese origin; in addition, there was also a cluster exclusive to Israel. In clade B, a specific cluster was identified that can be attributed to Fiji. For clades C and D, it is difficult to ascertain if there is stratification due to the lower number of genomes reconstructed for these two clades. While geographical stratification was evident for some intra-clade sub-types, most sub-types appeared to be multi-country and even multi-continental, indicating that *P. copri* is widely geographically distributed not only at the clade level but also at the intra-clade level.

Associating *P. copri* Clades with Metagenomically Investigated Human Diseases

A question that remains to be resolved is whether *P. copri* is beneficial or detrimental to human health, as studies report conflicting results (De Vadder et al., 2016; Dillon et al., 2014; Kovatcheva-Datchary et al., 2015; Pedersen et al., 2016; Scher et al., 2013; Wen et al., 2017). Here, in a meta-analysis of available disease phenotypes, we found no strong evidence that any of the four clades were associated with a disease. Specifically, to investigate the association of the *P. copri* complex with different diseases, we analyzed the prevalence and abundance of the four clades for each cohort where the study design included both case and controls. In total, there were ten datasets including colorectal cancer (CRC) (Feng et al., 2015; Vogtmann et al., 2016; Yu et al., 2017; Zeller et al., 2014), type 2 diabetes (T2D) (Karlsson et al., 2013; Qin et al., 2012), hypertension (Li et al., 2017), liver cirrhosis (Qin et al., 2014), and inflammatory bowel disease (IBD) (He et al., 2017; Nielsen et al., 2014).

To identify and estimate the abundance of each of *P. copri* clades within a sample, the metagenomic reads were mapped

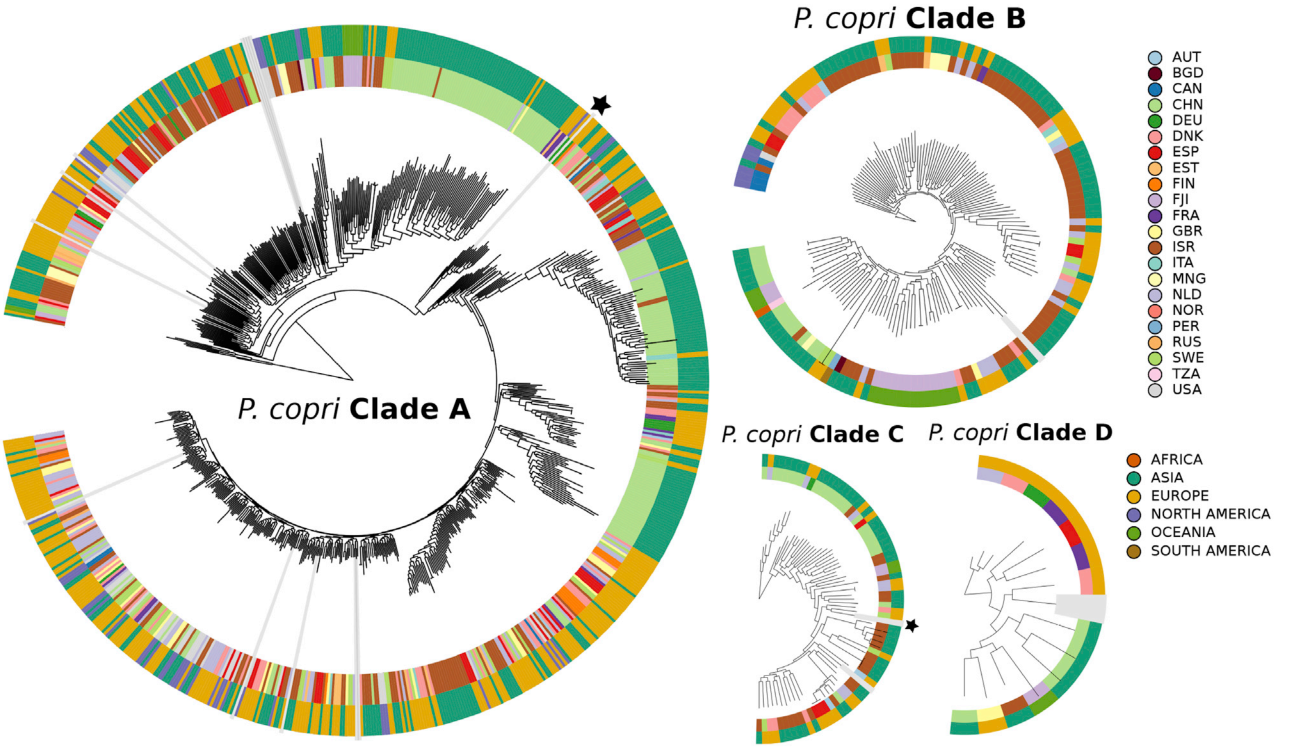

Figure 2. Phylogenetic Representation of All 1,023 *P. copri* Genomes Separated for Each Clade of the *P. copri* Complex
Outer ring is colored by continent of origin and inner ring is colored by country. Radial gray bars indicate recently sequenced isolate genomes, and publicly available reference genomes are denoted by black stars.

to a panel of unique clade-specific markers inferred for each of the four clades (see STAR Methods). The most significant changes in abundance and prevalence of *P. copri* and specifically the four clades were identified in the CRC and adenoma cohort of Feng et al. (2015), with both clades A and C being associated with disease (Figure S2A). However, three other CRC cohorts considered (Vogtmann et al., 2016; Yu et al., 2017; Zeller et al., 2014) and an overall CRC meta-analysis of seven cohorts failed to support this observation (Thomas et al., 2019). Generally, while there were some weak associations of the *P. copri* clades in disease, across the control samples of the different datasets, we observed heterogeneity in both abundance and prevalence suggesting significant batch effects. As such, at the clade level, there is no clear evidence to suggest *P. copri* is associated with the etiology of these diseases (Figure S2A). Extending the analysis further to consider sub-clades also did not reveal any statistically significant associations with disease (Figure S2B; see STAR Methods). Finally, we considered if the *P. copri* complex could be associated with other factors such as body mass index (BMI) or age (Table S1). Similar to disease, we note potential batch and cohort effects but no significant differences with all four clades being identified across all age groups and BMI categories.

Reconstruction of 98 Additional Genomes from Non-Westernized Samples Expands the Diversity of the *P. copri* Clades with Fewer Representatives

Most of our understanding of the microbiome has been accumulated from Westernized populations (Brewster et al., 2019). While small in comparison a number of public datasets have been generated from non-Westernized populations, which were included in the above analysis. These datasets sampled individuals inhabiting Peru (Obregon-Tito et al., 2015), Fiji (Brito et al., 2016), and Mongolia (Liu et al., 2016) and two datasets from Tanzania (Rampelli et al., 2015; Smits et al., 2017) totaling 340 metagenomes. The term "Westernization" encompasses many factors including lifestyle, environment, and diet (for full description, see STAR Methods). A common feature of non-Westernized datasets is a high *Prevotella* prevalence (De Filippo et al., 2010; Hansen et al., 2019; Obregon-Tito et al., 2015; Schnorr et al., 2014; Smits et al., 2017; Yatsunenko et al., 2012) and particularly *P. copri* (Pasolli et al., 2019; Vangay et al., 2018). To further investigate the prevalence and abundance of *P. copri* in non-Westernized populations, we also considered our recently sequenced dataset of non-Westernized adults from Madagascar (110 metagenomes) (Pasolli et al., 2019) and three additional non-Westernized cohorts sequenced in this work. These included paired infant and mother samples from Ethiopia (50 metagenomes) and extended families from Ghana and Tanzania (44 and 68 metagenomes, respectively) (see STAR Methods). From these additional 272 metagenomes (Table S2), we reconstructed 98 high quality *P. copri* complex genomes expanding the clades with fewer reconstructed members (clade A, B, C, and D were expanded by 3.4%, 34.7%, 17.6%, and 40%, respectively) (Figure S3; Table S2). An additional feature of three of our recently sequenced datasets was that they included sampling within families. This offered the potential to establish

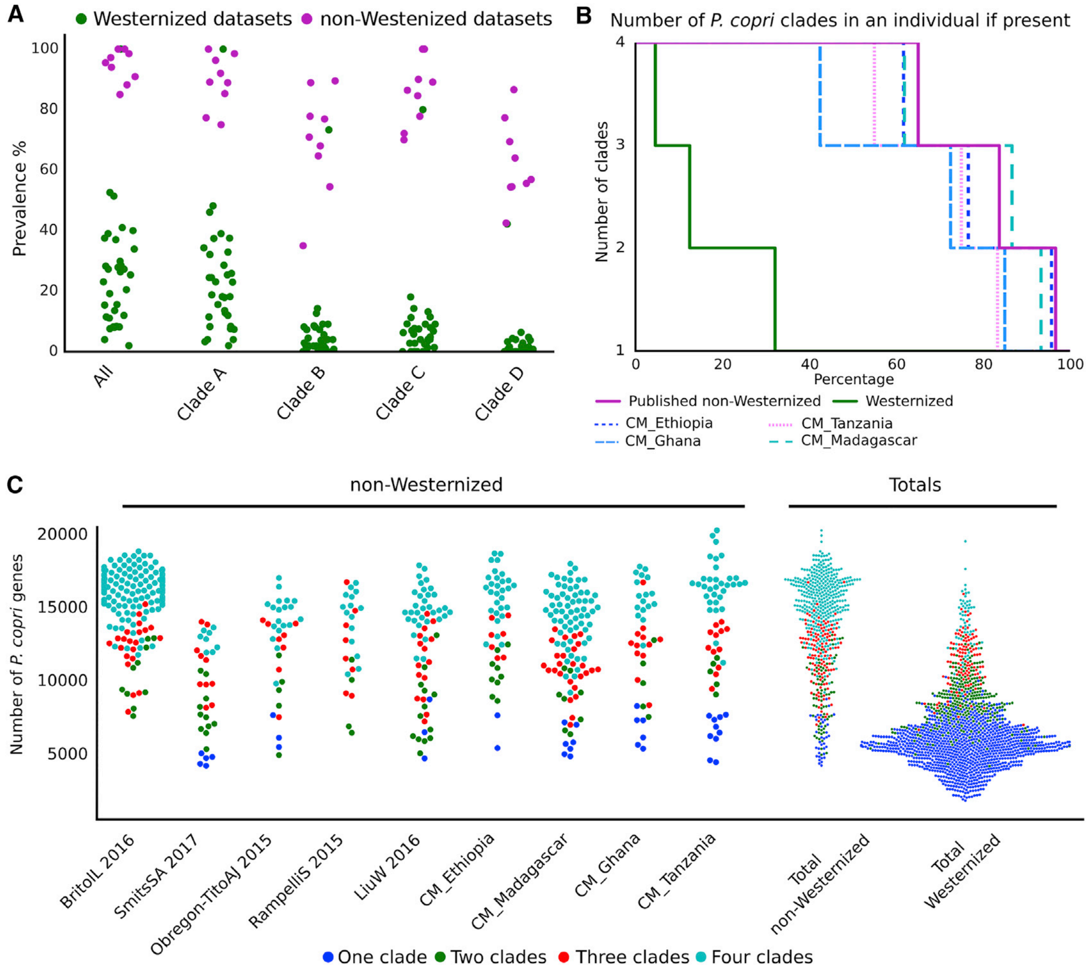

Figure 3. Prevalence of the *P. copri* Complex and Its Association with Non-Westernized Populations

(A) *P. copri* prevalence in non-Westernized and Westernized datasets. "All" refers to the prevalence of any of the four clades being present.

(B) Percentage of individuals harboring multiple *P. copri* clades.

(C) *P. copri* complex pangenome sizes for non-Westernized individuals by dataset compared to Westernized individuals.

if transmission occurs and, if so, to what extent within families. When *P. copri* genomes were reconstructed from more than one family member, we compared the genetic distances to estimate the level of intra-family strain sharing. Using normalized phylogenetic tree distances and cutoffs proposed previously (Truong et al., 2017) (see STAR Methods), in 5 of 26 cases (19.2%), we identified the same strain, suggesting possible horizontal and/or vertical transmission of the *P. copri* complex within families; therefore, the familial prevalence of *P. copri* could potentially be an important source in acquisition.

Co-presence of Multiple *P. copri* Complex Clades Is Typical in Individuals from Non-Westernized Populations

We detected the presence of the *P. copri* complex in all 40 datasets considered, but the prevalence in non-Westernized populations was nearly ubiquitous (95.4% prevalence), in contrast to Westernized populations (29.6% prevalence) (Figure 3A; Table S2). Considering each clade separately, all four were significantly more prevalent in non-Westernized compared to westernized datasets (p values < 1.1e-12, Welch's t test), with clade A being the most prevalent (91.5% in non-Westernized versus 26.9% in Westernized populations) followed by C (88.2% versus 8.35%), B (73.5% versus 6.2%), and D (68.8% versus 2.7%, Figure 3A). The finding that all four *P. copri* clades are always higher in non-Westernized populations spanning multiple countries and continents than Westernized populations is remarkable, with the only exception being Mongolia (Liu et al., 2016). The Mongolian cohort sampled both urban dwellers and rural non-Westernized populations. While the urban dwellers have a prevalence closer to that of the non-Westernized populations (clade A prevalence, 100%; B, 73.3%; C, 80%; and D, 42.2%), this is still generally lower than the rural non-Westernized Mongolian population (clade A prevalence, 98.5%; B, 76.9%; C, 84.6%; and D, 56.9%). Although *P. copri* was observed at a much lower prevalence in Westernized populations, all four clades were detected, and of those, clade A was the most prevalent type (Figure 3A).

Considering the high prevalence of the four *P. copri* clades in non-Westernized populations, we next sought to identify if these

clades are mutually exclusive or able to co-inhabit in the intestine. Analysis of our sequenced datasets clearly revealed multiple clades being present within non-Westernized individuals (Figure S4A) and confirmed in other non-Westernized datasets (Figure 3B). Strikingly, for the 95.4% of non-Westernized individuals with at least one *P. copri* complex clade, in 61.6% of these, all four clades were detectable; in 82.0%, at least three; and at least two in 93.8% of individuals. The high percentage of individuals carrying multiple clades was a consistent feature observed across all non-Westernized datasets spanning four continents (Figure 3B). In comparison, in the smaller fraction (29.6%) of Westernized individuals with at least one *P. copri* complex clade, only 4.6% had all four clades; 12.5%, at least three; and 32.1%, more than one. Therefore, we demonstrate that not only is *P. copri* prevalence higher in non-Westernized populations, but the pattern of multi-clade co-presence in these populations is also a defining characteristic.

Due to the existence of multiple clades within an individual (Figures 3B and S4A) and the observation of a sizable inter-clade diversity based on gene content (Figure 1D), we decided to estimate the sum of unique *P. copri* complex genes within each individual or rather "the within individual *P. copri* pangenome" (see STAR Methods). As expected, individuals with multiple clades tended toward a larger number of unique *P. copri* complex genes (Figure 3C), and as multiple clades is a feature of being non-Westernized, a considerably larger *P. copri* functional potential was revealed in these populations (Figures 3C and S4B).

Evidence of Distinct Carbohydrate Metabolism Repertoires in the Four *P. copri* Complex Clades

To investigate the functional diversity of the *P. copri* complex, we annotated the open reading frames (ORFs) for each genome using the eggNOG database (Huerta-Cepas et al., 2017) (see STAR Methods). Between and also within the four clades of the complex, we observed considerable functional diversity, with clade B being the most dissimilar based on the overall distance of the eggNOG functional profiles (Figure 4A), which is consistent with the inter-clade genetic diversity observed above (Figures 1B–1D). Some of the distinguishing functionalities included sulfur metabolism and assimilation, which were enriched in all clades relative to B (Table S3). Similarly, in carbohydrate metabolism, β-galactosidase was found to be absent in clade B while being relatively common in all other clades (at least present in >60% of genomes). In the metabolism of cofactors and vitamins, genes responsible for folate metabolism were depleted in clade D. Interestingly, clade D also had the least diversity of antimicrobial resistance genes lacking 5 out of 7 identified in the other three clades. Differences were also noticeable in membrane transporters; for instance, the polyamine spermidine/putrescine ABC transporter (pot*ABCD*) was present in almost all members of clades A, C, and D but never observed in clade B. Conversely, an energy coupling factor (ECF)-type ABC transporter that could be responsible for micronutrient uptake was solely found in a subset of genomes of clade B (27% of genomes).

One reported feature of *P. copri* is its effect on glucose homeostasis (De Vadder et al., 2016; Kovatcheva-Datchary et al., 2015; Pedersen et al., 2016), with one recent study suggesting a positive benefit via succinate production (De Vadder et al., 2016). While potential succinate production was observed in all four clades the genes responsible were less prevalent in clade B (p value < 5.2e-37, Bonferroni-corrected Fisher's exact test). On the contrary, high levels of circulating branched chain amino acids (BCAAs), linked to the development of insulin resistance, have been associated with higher levels of *P. copri* in the gut microbiome (Pedersen et al., 2016). In addition, the presence of genes for BCAA biosynthesis in the *P. copri* pangenome has been shown to be diet dependent and associated with actual urinary BCAA levels (De Filippis et al., 2019). Here, we found that BCAA biosynthesis genes were widespread and not significantly associated with any given clade (>85% present in all clades).

The *P. copri* complex is strongly associated with non-Westernized populations that have diets that are typically higher in fiber and complex carbohydrates and lower in fats and animal protein than typical Western diets (De Filippo et al., 2010; Segata, 2015; Statovci et al., 2017). To specifically look at the *P. copri* complex for potential carbohydrate utilization, the genomes were also screened for carbohydrate active enzymes (CAZymes) (Lombard et al., 2014) (see STAR Methods). While many of these CAZy families were found to be common to all four *P. copri* clades (Table S3), considerable variability in the presence of these families was observed between and even within the different clades (Figures 4B, 4C, and S4C). To focus on families potentially associated with plant-derived carbohydrate degradation (e.g., cellulose, hemicellulose, and pectin), each family was ascribed a broad substrate specificity via manual curation (Table S3). While all clades were found to have the potential to degrade plant-derived carbohydrates, not all CAZy families were represented or equally distributed throughout the four clades (Table S3); for example, the polysaccharide pectin-degrading families PL9 and PL10 were highly prevalent and nearly exclusive to clade B. In clade D, the GH9 CAZy family of cellulases was particularly enriched compared to the other clades (Figure 4C). The distinct clustering based on CAZy gene content (Figure 4B) displaying inter- and intra-clade functional differences suggests overlapping but potential heterogeneity in carbohydrate metabolism. The frequent co-presence of all four clades in non-Westernized populations would suggest that they are non-competing and therefore niche separated. While it cannot be discounted that these four clades are spatially separated in the intestine, the ability to utilize a differing array of carbohydrates could potentially be the driver of this separation. Within an individual, the presence of multiple clades collectively offers a larger and perhaps complementary functionality to efficiently metabolize a wide range of dietary carbohydrates.

P. copri Diversity in Ancient Human Gut Contents Resembles that of Non-Westernized Populations and Gives Insights into Its Evolutionary History

To ascertain if the high *P. copri* prevalence and co-presence of the four clades in non-Westernized populations reflects the composition in ancient human gut microbiomes, we analyzed the gut content of four archaeological samples. We studied material from the lower intestinal tract and lung tissue of the Iceman, a 5,300-year-old natural ice mummy (Spindler, 1994). The Iceman genetically belongs to the Early European Farmers and originated and lived in Southern Europe, in the Eastern Italian

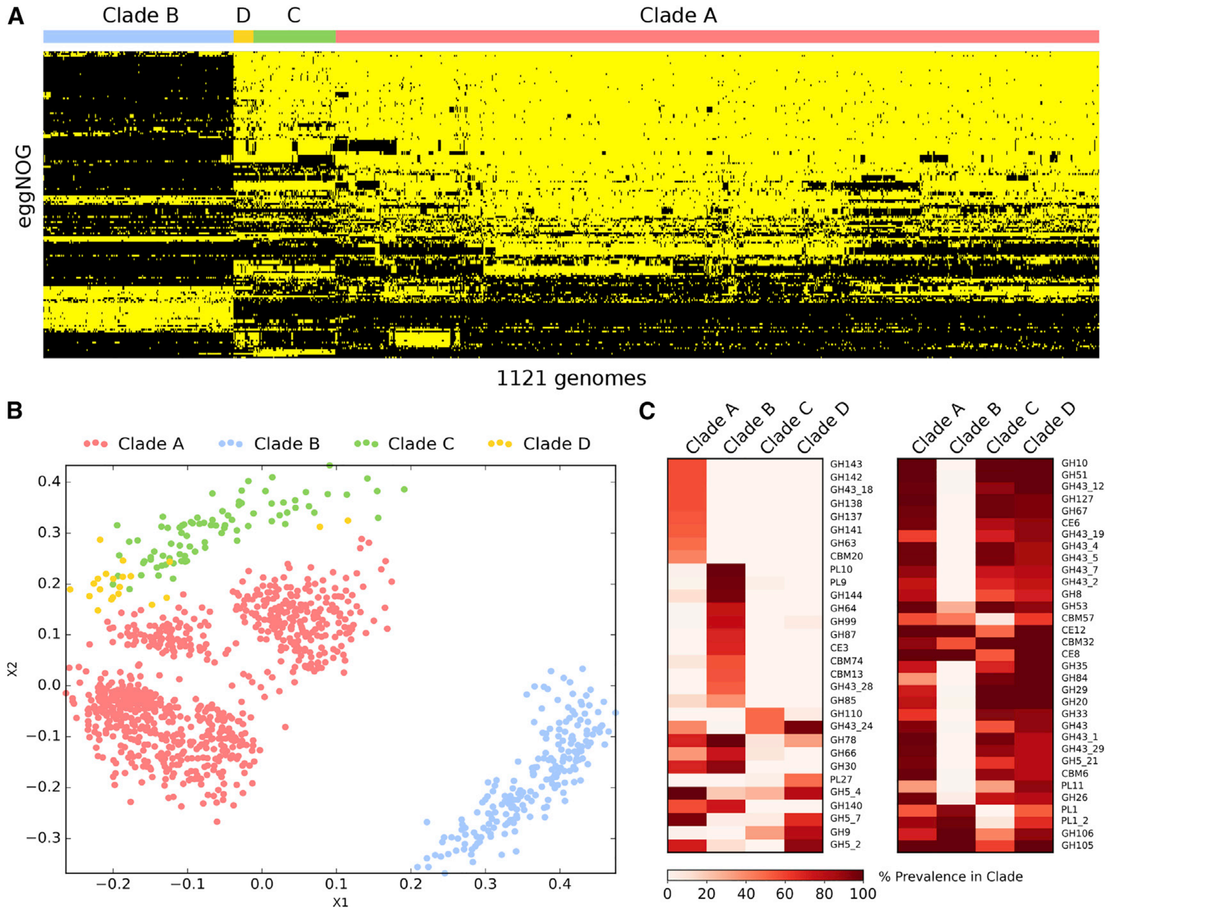

Figure 4. Functional Diversity of the *P. copri* Complex

(A) Presence and absence of eggNOG functions significantly different between the four *P. copri* clades (yellow, present; black, absent) (see STAR Methods).

(B) Multidimensional scaling (MDS) ordination based on CAZy families present in each genome showing distinct inter- and intra-clustering in the *P. copri* complex.

(C) All CAZy families significantly enriched (left) or depleted (right) in at least one clade relative to each of the other three (see STAR Methods). Prevalence is defined as the percentage of genomes in that clade for which at least one gene belongs to the given CAZy family. For full list of CAZy prevalence in each clade, see Table S3.

Alps (Haak et al., 2015; Keller et al., 2012; Lazaridis et al., 2014; Müller et al., 2003) (Figure 5A). We also analyzed three coprolite samples (fossilized feces) (Figure 5A) recovered from the pre-Columbian (1,300 ± 100 BP) site "La Cueva de los Muertos Chiquitos" from Durango, a Northwestern state of Mexico (Brooks et al., 1962) (see STAR Methods).

We found the *P. copri* complex to be present in both the Mexican and the European ancient gut metagenomes (Figure 5B). All samples had at least two *P. copri* clades (clades A and C), and in two coprolites, all four clades could be detected. The higher prevalence of clade A and C in our ancient samples mirrors the tendency of modern-day populations (both Westernized and non-Westernized) where these two clades are more prevalent (Figure 3A). To discount the possibility of a non-ancient gut origin, we verified that the *P. copri* reads displayed damage patterns indicative of ancient DNA (Figures S5A and S5B) (Orlando et al., 2015), and in a control sample (Iceman lung tissue), no *P. copri* clades were detected (positive for only a single marker of the 2,448 *P. copri* complex specific markers) (Figure 5B). Two characteristics point toward a similarity between the ancient samples and modern non-Westernized populations. First, *P. copri* is common in the ancient samples like non-Westernized samples. Second, the ancient samples are characterized by a high clade co-presence (presence of 2 to 4 distinct clades) as observed in non-Westernized individuals. While we have only analyzed a small number of ancient metagenomes, we show that the likelihood of observing a high co-presence in the non-Westernized samples by chance is very low (Figure S5C). Together, the similarities between ancient and contemporary non-Westernized individuals suggest that the *P. copri* carriage pattern in non-Western populations is more akin to our ancestors.

To calibrate a *P. copri* phylogeny, we screened all ancient samples and found that one coprolite (sample 2180, radiocarbon dated AD 673 to 768, see STAR Methods) had sufficiently high coverage of clade A to be used for tip calibration (see STAR Methods). Model selection indicated that this dataset is best modeled by a strict clock suggesting a constant rate of evolution through time and in different *P. copri* clades (see STAR Methods). All our divergence estimates converged satisfactorily on clear posterior means (Figure 5C) and the age estimates

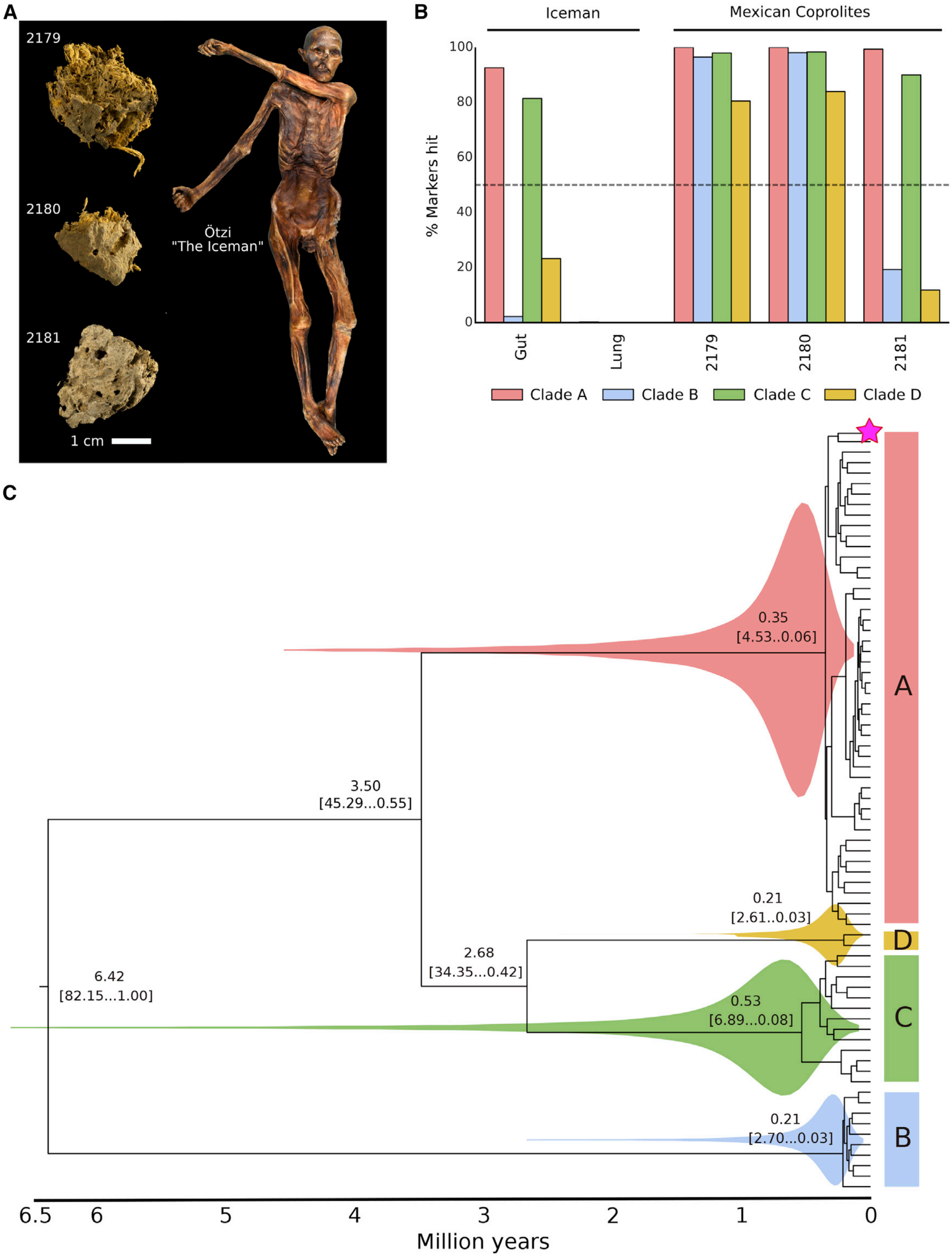

Figure 5. Ancient Microbiomes and the Evolutionary History of the *P. copri* Complex

(A) Ancient Mexican coprolite samples and intestinal and lung tissue sampled from the Iceman, a natural ice mummy.

(B) Percentage of positive *P. copri* clade-specific markers identified in each ancient metagenomic sample.

(C) Time-resolved phylogenetic tree of the *P. copri* complex; magenta star indicates the ancient coprolite sample, 2180 (see STAR Methods).

indicate that *P. copri* began to diversify (split of clade B) ~6.5 million years ago (Figure 5C). The diversification of clades A, D, and C is estimated to have occurred between ~3.5 and ~2.5 million years ago (Ungar and Sponheimer, 2011; Wood and Collard, 1999). The differentiation within each of the clades is instead relatively recent with the median estimates following that of the emergence of *Homo sapiens* circa 315 ka (Hublin et al., 2017). Despite the range in estimated clade divergence, even at the lowest estimation (420 ka), this occurred well before the first human migration waves out of Africa circa 90–194 ka years ago (Grün et al., 2005; Hershkovitz et al., 2018). This would indicate that the four clades of the *P. copri* complex were a feature of our pre-migratory human ancestors.

Further support of *P. copri* clade diversification prior to migration is that the high prevalence of all four clades and clade co-presence within an individual is a consistent feature of disparate non-Westernized populations in Africa, Oceania, South America, and Asia. This, together with the estimation of clade divergence, implies the *P. copri* complex has been a long-standing feature of the human microbiome. This analysis and the observed multi-generational decrease in the prevalence of *Prevotella* strains in non-Western migrants upon Westernization (Vangay et al., 2018) suggests that the underrepresentation of *P. copri* in Westernized populations could be due to its loss in response to Westernization. This loss has rapidly occurred in an almost infinitesimal time frame relative to host-microbe coevolution.

DISCUSSION

We demonstrate that *P. copri* is not a monotypic species but four clearly defined clades, each spanning a diversity that is typical of species (Figure 1), and all four clades have the potential to reside either solely or in combination within an individual (Figure 3B). We propose to name this group the *P. copri* complex, comprising clades A, B, C, and D. The insights that we have gained into the *P. copri* complex genetics and population genomics relied on isolate sequencing, on sequencing individuals from underrepresented non-Westernized populations, and largely on the tremendous resource of publicly available metagenomic datasets, covering multiple countries, diseases, and lifestyles. This led to the observation that the *P. copri* complex is globally distributed (Figure 2), but with a highly structured distribution, both in terms of prevalence and the presence of multiple clades within an individual in non-Westernized populations (Figure 3).

While we concede that the term Westernized versus non-Westernized serves to demarcate what may be better seen as a continuum along multiple lifestyle parameters, the distinction nonetheless has its merits, as interest grows in comparing Westernized microbiomes to those presumed more akin to our ancestral microbiomes. Recent studies have expanded our understanding of the microbial diversity of non-Westernized populations (Hansen et al., 2019; Pasolli et al., 2019) and the rapid loss of diversity with Westernization (Vangay et al., 2018). What is still to be determined are the consequences of this microbial impoverishment with respect to the wider gut microbial ecosystem and its impact on human health.

Evidence from the analysis of ancient stool samples (Figure 5) suggests that *P. copri* diverged into four clades prior to the first human migration events out of Africa. The fact that we consistently observe high prevalence in globally disparate non-Westernized populations and in ancient microbiomes suggests that the loss of *P. copri* might be a result of Westernization. A major element of Westernization has been a shift in diet over the course of the last two centuries with the advent of industrialization and food processing, from one typically high in fiber and complex carbohydrates to one high in sodium, fat, and simple sugars and low in fiber. It was previously shown that *P. copri* provides a host benefit in response to a high-fiber diet (De Vadder et al., 2016; Kovatcheva-Datchary et al., 2015) but not one high in fat (Pedersen et al., 2016). The *P. copri* complex shows a diversity in plant-derived carbohydrate utilization (Figure 4), which may suggest that diet is a key driver responsible for its ultimate demise in Westernized populations.

Diet in particular seems to play a pivotal role in the case of *P. copri*, yet it is extremely difficult to study this influence in the context of long-term human dietary modifications spanning multiple generations. Given previous work associating multi-generational microbial impoverishment with dietary changes in mice (Sonnenburg et al., 2016), clearly more work is required both *in silico* and using *in vitro* studies to functionally associate and characterize the *P. copri* complex with respect to long-term dietary exposures, transmission, and retention. In part, *P. copri* has come to attention based on its association with disease, but in this study, we found no clear evidence that particular clades are associated with the subset of health conditions available for meta-analysis. Nevertheless, it cannot be disregarded that such associations may exist, possibly only at the sub-clade level, but such an investigation would require the power of a far larger number of disease-specific cohorts than are currently available.

Finally, it is particularly notable that the analysis approach taken here is generalizable to other microbial species in instances where there are minimal reference isolate sequences available. This is, in principle, the case for species that are understudied due to being recalcitrant to cultivation or because they have not been the focus of sequencing efforts. The ever-increasing number of publicly available metagenomes will serve this end, as well as likely add clarity to whether *P. copri* is considered either a positive or a negative influence on health in the context of other microbiome members, diet, lifestyle, and host genetic factors. This study reveals that *P. copri* is far more complex than previously imagined, and it will be important in future studies to appreciate this in order not to oversimplify and underestimate the potential *P. copri* diversity within the human gut microbiome.

STAR★METHODS

Detailed methods are provided in the online version of this paper and include the following:

- KEY RESOURCES TABLE
- LEAD CONTACT AND MATERIALS AVAILABILITY
- EXPERIMENTAL MODEL AND SUBJECT DETAILS
- METHOD DETAILS
  - Metagenomic Assembly
  - Constructing a *P. copri* Genome Panel with Additional Sequenced Isolates and Manually Curated Genomes from Metagenomes

- ○ Automated Reconstruction of *P. copri* Genomes from >6500 Metagenomes
- ○ *P. copri* Genome Quality Control
- ○ Genetic Distance between and within the *P. copri* Complex and Related Species
- ○ Phylogenetic Analysis
- ○ The *P. copri* Pangenome and Evaluating Prevalence and Abundance
- ○ Westernisation and Additional Non-Westernized Datasets
- ○ Genome Functional Potential Analysis
- ○ Inferring *P. copri* Sub-clades Based on Function
- ○ Iceman Samples and Mexican Coprolite Material
- ○ *P. copri* Genome Reconstruction from Ancient Gut Metagenomes and Molecular Dating
- QUANTIFICATION AND STATISTICAL ANALYSIS
- DATA AND CODE AVAILABILITY

SUPPLEMENTAL INFORMATION

Supplemental Information can be found online at https://doi.org/10.1016/j.chom.2019.08.018.

ACKNOWLEDGMENTS

We thank Levi Waldron and the members of his laboratory for the effort in developing and supporting *curatedMetagenomicData* (Pasolli et al., 2017) and all the members of the Segata laboratory for fruitful discussions and support. We also thank Eleonora Nigro for her illustration of the visual abstract. We thank the team of the NGS Core Facility at CIBIO (Veronica De Sanctis, Roberto Bertorelli, and Paola Fassan) for support in sample preparation and for metagenomic sequencing and the high-performance computing team at the University of Trento. We thank the GeNaPi Project, a collaborative study between Emalaikat Foundation, University of Valencia, and IATA-CSIC, for data and sample collection from the Ethiopia cohort, particularly Mari Olcina and Lourdes Larruy. We thank all field workers and laboratory technicians involved in the sample collection in Ghana and Tanzania. This project has received funding from the European Research Council (ERC) under the European Union's Horizon 2020 research and innovation programme (grant agreement No 716575) to N.S. This work was also supported by the NIH NHGRI grant R01HG005220, the NIDDK grant R24DK110499, the NIDDK grant U54DE023798, and the CMIT grant 6935956 to C.H.; the NYU-HHC CTSI grant (1TL1TR001447) to H.F.P.; the US National Institute of Health grant R01-DK103358-01 to R.B. and D.R.L.; the Howard Hughes Medical Institute to D.R.L.; and the MIUR "Futuro in Ricerca" RBFR13EWWI_001, the European Union (H2020-SFS-2018-1 project MASTER-818368 and H2020-SC1-BHC project ONCOBIOME-825410) to N.S. Further support was provided by the Programma Ricerca Budget Eurac 2017 of the Province of Bolzano, Italy to F.M., and by the EU-H2020 (DiMeTrack-707345) to E.P. and N.S.

AUTHOR CONTRIBUTIONS

Conception and Design, A.T. and N.S.; Sample Collection, Processing, and Data generation, H.F.P., F. Armanini, K.B., C.M., R.B., J.L., J.A., K.R., T.R., F.B., L.E., A.Z., M.C.C., D.R.L., D.E., and F.M.; Public Data Collection and Curation, A.T., E.P., N.K., and P.M.; Data Analysis; A.T., E.P., K.D.H, F. Asnicar, N.K., M.Z., F.D.F., D.E., O.R.S., and F.M.; Data Interpretation, A.T., K.D.H., F.D.F., D.E., O.R.S., C.H., F.M., and N.S.; Writing – Original Draft, A.T. and N.S.; Writing – Review & Editing, A.T., N.S., C.H., O.R.S., D.E., and F.M. All authors read and approved the final manuscript.

DECLARATION OF INTERESTS

The authors declare no competing interests.

Received: April 15, 2019
Revised: July 5, 2019
Accepted: August 28, 2019
Published: October 10, 2019

SUPPORTING CITATIONS

The following references appear in the Supplemental Information: Asnicar et al. (2017); Bäckhed et al. (2015); Bengtsson-Palme et al. (2015); Ferretti et al. (2018); Gevers et al. (2014); Hannigan et al. (2018); Kostic et al. (2015); David et al. (2015); Le Chatelier et al. (2013); Li et al. (2014); Li et al. (2016); Loman et al. (2013); Louis et al. (2016); Raymond et al. (2016); Schirmer et al. (2016); Vatanen et al. (2016); Vincent et al. (2016); Xie et al. (2016); Zeevi et al. (2015).

REFERENCES

Achilli, A., Perego, U.A., Bravi, C.M., Coble, M.D., Kong, Q.P., Woodward, S.R., Salas, A., Torroni, A., and Bandelt, H.J. (2008). The phylogeny of the four Pan-American MtDNA haplogroups: implications for evolutionary and disease studies. PLoS One *3*, e1764.

Altschul, S.F., Gish, W., Miller, W., Myers, E.W., and Lipman, D.J. (1990). Basic local alignment search tool. J. Mol. Biol. *215*, 403–410.

Andrews, R.M., Kubacka, I., Chinnery, P.F., Lightowlers, R.N., Turnbull, D.M., and Howell, N. (1999). Reanalysis and revision of the Cambridge reference sequence for human mitochondrial DNA. Nat. Genet. *23*, 147.

Asnicar, F., Manara, S., Zolfo, M., Truong, D.T., Scholz, M., Armanini, F., Ferretti, P., Gorfer, V., Pedrotti, A., Tett, A., et al. (2017). Studying Vertical Microbiome Transmission from Mothers to Infants by Strain-Level Metagenomic Profiling. mSystems *2*, e00164-16.

Asnicar, F., Weingart, G., Tickle, T.L., Huttenhower, C., and Segata, N. (2015). Compact graphical representation of phylogenetic data and metadata with GraPhlAn. PeerJ *3*, e1029.

Bäckhed, F., Roswall, J., Peng, Y., Feng, Q., Jia, H., Kovatcheva-Datchary, P., Li, Y., Xia, Y., Xie, H., Zhong, H., et al. (2015). Dynamics and Stabilization of the Human Gut Microbiome during the First Year of Life. Cell Host Microbe *17*, 690–703.

Bengtsson-Palme, J., Angelin, M., Huss, M., Kjellqvist, S., Kristiansson, E., Palmgren, H., Larsson, D.G.J., and Johansson, A. (2015). The Human Gut Microbiome as a Transporter of Antibiotic Resistance Genes between Continents. Antimicrob. Agents Chemother. *59*, 6551–6560.

Bodner, M., Perego, U.A., Huber, G., Fendt, L., Röck, A.W., Zimmermann, B., Olivieri, A., Gómez-Carballa, A., Lancioni, H., Angerhofer, N., et al. (2012). Rapid coastal spread of first Americans: novel insights from South America's Southern Cone mitochondrial genomes. Genome Res. *22*, 811–820.

Bouckaert, R., Heled, J., Kühnert, D., Vaughan, T., Wu, C.H., Xie, D., Suchard, M.A., Rambaut, A., and Drummond, A.J. (2014). BEAST 2: a software platform for Bayesian evolutionary analysis. PLoS Comput. Biol. *10*, e1003537.

Bowers, R.M., Kyrpides, N.C., Stepanauskas, R., Harmon-Smith, M., Doud, D., Reddy, T.B.K., Schulz, F., Jarett, J., Rivers, A.R., Eloe-Fadrosh, E.A., et al. (2017). Minimum information about a single amplified genome (MISAG) and a metagenome-assembled genome (MIMAG) of bacteria and archaea. Nat. Biotechnol. *35*, 725–731.

Bressert, E. (2012). SciPy and NumPy: an Overview for Developers (O'Reilly Media, Inc.).

Brewster, R., Tamburini, F.B., Asiimwe, E., Oduaran, O., Hazelhurst, S., and Bhatt, A.S. (2019). Surveying gut microbiome research in Africans: toward improved diversity and representation. Trends Microbiol.

Brito, I.L., Yilmaz, S., Huang, K., Xu, L., Jupiter, S.D., Jenkins, A.P., Naisilisili, W., Tamminen, M., Smillie, C.S., Wortman, J.R., et al. (2016). Mobile genes in the human microbiome are structured from global to individual scales. Nature *535*, 435–439.

Brooks, R.H., Kaplan, L., Cutler, H.C., and Whitaker, T.W. (1962). Plant material from a cave on the Rio zape, Durango, Mexico. Am. antiq. *27*, 356–369.

Buchfink, B., Xie, C., and Huson, D.H. (2015). Fast and sensitive protein alignment using DIAMOND. Nat. Methods *12*, 59–60.

Camacho, M., Araújo, A., Morrow, J., Buikstra, J., and Reinhard, K. (2018). Recovering parasites from mummies and coprolites: an epidemiological approach. Parasit. Vectors *11*, 248.

Cani, P.D. (2018). Human gut microbiome: hopes, threats and promises. Gut *67*, 1716–1725.

Capella-Gutiérrez, S., Silla-Martínez, J.M., and Gabaldón, T. (2009). trimAl: a tool for automated alignment trimming in large-scale phylogenetic analyses. Bioinformatics *25*, 1972–1973.

David, L.A., Weil, A., Ryan, E.T., Calderwood, S.B., Harris, J.B., Chowdhury, F., Begum, Y., Qadri, F., LaRocque, R.C., and Turnbaugh, P.J. (2015). Gut microbial succession follows acute secretory diarrhea in humans. MBio *6*, e00381-15.

De Filippis, F., Pasolli, E., Tett, A., Tarallo, S., Naccarati, A., De Angelis, M., Neviani, E., Cocolin, L., Gobbetti, M., Segata, N., et al. (2019). Distinct genetic and functional traits of human intestinal Prevotella copri strains are associated with different habitual diets. Cell Host Microbe *25*, 444–453.e3.

De Filippo, C., Cavalieri, D., Di Paola, M., Ramazzotti, M., Poullet, J.B., Massart, S., Collini, S., Pieraccini, G., and Lionetti, P. (2010). Impact of diet in shaping gut microbiota revealed by a comparative study in children from Europe and rural Africa. Proc. Natl. Acad. Sci. USA *107*, 14691–14696.

De Vadder, F., Kovatcheva-Datchary, P., Zitoun, C., Duchampt, A., Bäckhed, F., and Mithieux, G. (2016). Microbiota-produced succinate improves glucose homeostasis via intestinal gluconeogenesis. Cell Metab. *24*, 151–157.

Dillon, S.M., Lee, E.J., Kotter, C.V., Austin, G.L., Dong, Z., Hecht, D.K., Gianella, S., Siewe, B., Smith, D.M., Landay, A.L., et al. (2014). An altered intestinal mucosal microbiome in HIV-1 infection is associated with mucosal and systemic immune activation and endotoxemia. Mucosal Immunol. *7*, 983–994.

Eddy, S.R. (2011). Accelerated profile HMM Searches. PLoS Comput. Biol. *7*, e1002195.

Eren, A.M., Esen, Ö.C., Quince, C., Vineis, J.H., Morrison, H.G., Sogin, M.L., and Delmont, T.O. (2015). Anvi'o: an advanced analysis and visualization platform for 'omics data. PeerJ *3*, e1319.

Ermini, L., Olivieri, C., Rizzi, E., Corti, G., Bonnal, R., Soares, P., Luciani, S., Marota, I., De Bellis, G., Richards, M.B., et al. (2008). Complete mitochondrial genome sequence of the Tyrolean Iceman. Curr. Biol. *18*, 1687–1693.

Fehlner-Peach, H., Magnabosco, C., Raghavan, V., Scher, J.U., Tett, A., Coz, L.M., et al. (2019). Distinct polysaccharide growth profiles of human intestinal Prevotella copri isolates. bioRxiv. https://doi.org/10.1101/750802.

Fehren-Schmitz, L., Llamas, B., Lindauer, S., Tomasto-Cagigao, E., Kuzminsky, S., Rohland, N., Santos, F.R., Kaulicke, P., Valverde, G., Richards, S.M., et al. (2015). A re-appraisal of the early Andean human remains from Lauricocha in Peru. PLoS One *10*, e0127141.

Feng, Q., Liang, S., Jia, H., Stadlmayr, A., Tang, L., Lan, Z., Zhang, D., Xia, H., Xu, X., Jie, Z., et al. (2015). Gut microbiome development along the colorectal adenoma-carcinoma sequence. Nat. Commun. *6*, 6528.

Ferretti, P., Pasolli, E., Tett, A., Asnicar, F., Gorfer, V., Fedi, S., Armanini, F., Truong, D.T., Manara, S., Zolfo, M., et al. (2018). Mother-to-Infant Microbial Transmission from Different Body Sites Shapes the Developing Infant Gut Microbiome. Cell Host Microbe *24*, 133–145.e5.

Forouzan, E., Shariati, P., Mousavi Maleki, M.S., Karkhane, A.A., and Yakhchali, B. (2018). Practical evaluation of 11 de novo assemblers in metagenome assembly. J. Microbiol. Methods *151*, 99–105.

Gevers, D., Kugathasan, S., Denson, L.A., Vázquez-Baeza, Y., Van Treuren, W., Ren, B., Schwager, E., Knights, D., Song, S.J., Yassour, M., et al. (2014). The treatment-naive microbiome in new-onset Crohn's disease. Cell Host Microbe *15*, 382–392.

Gómez-Carballa, A., Catelli, L., Pardo-Seco, J., Martinón-Torres, F., Roewer, L., Vullo, C., and Salas, A. (2015). The complete mitogenome of a 500-year-old inca child mummy. Sci. Rep. *5*, 16462.

Goris, J., Konstantinidis, K.T., Klappenbach, J.A., Coenye, T., Vandamme, P., and Tiedje, J.M. (2007). DNA-DNA hybridization values and their relationship to whole-genome sequence similarities. Int. J. Syst. Evol. Microbiol. *57*, 81–91.

Grün, R., Stringer, C., McDermott, F., Nathan, R., Porat, N., Robertson, S., Taylor, L., Mortimer, G., Eggins, S., and McCulloch, M. (2005). U-series and ESR analyses of bones and teeth relating to the human burials from Skhul. J. Hum. Evol. *49*, 316–334.

Haak, W., Lazaridis, I., Patterson, N., Rohland, N., Mallick, S., Llamas, B., Brandt, G., Nordenfelt, S., Harney, E., Stewardson, K., et al. (2015). Massive migration from the steppe was a source for Indo-European languages in Europe. Nature *522*, 207–211.

Hammerl, E.E., Baier, M.A., and Reinhard, K.J. (2015). Agave chewing and dental wear: evidence from quids. PLoS One *10*, e0133710.

Hannigan, G.D., Duhaime, M.B., Ruffin, M.T., 4th, Koumpouras, C.C., and Schloss, P.D. (2018). Diagnostic Potential and Interactive Dynamics of the Colorectal Cancer Virome. MBio *9*, e02248-18.

Hansen, M.E.B., Rubel, M.A., Bailey, A.G., Ranciaro, A., Thompson, S.R., Campbell, M.C., Beggs, W., Dave, J.R., Mokone, G.G., Mpoloka, S.W., et al. (2019). Population structure of human gut bacteria in a diverse cohort from rural Tanzania and Botswana. Genome Biol. *20*, 16.

Hayashi, H., Shibata, K., Sakamoto, M., Tomita, S., and Benno, Y. (2007). Prevotella copri sp. nov. and Prevotella stercorea sp. nov., isolated from human faeces. Int. J. Syst. Evol. Microbiol. *57*, 941–946.

He, Q., Gao, Y., Jie, Z., Yu, X., Laursen, J.M., Xiao, L., Li, Y., Li, L., Zhang, F., Feng, Q., et al. (2017). Two distinct metacommunities characterize the gut microbiota in Crohn's disease patients. GigaScience *6*, 1–11.

Hershkovitz, I., Weber, G.W., Quam, R., Duval, M., Grün, R., Kinsley, L., Ayalon, A., Bar-Matthews, M., Valladas, H., Mercier, N., et al. (2018). The earliest modern humans outside Africa. Science *359*, 456–459.

Hublin, J.J., Ben-Ncer, A., Bailey, S.E., Freidline, S.E., Neubauer, S., Skinner, M.M., Bergmann, I., Le Cabec, A., Benazzi, S., Harvati, K., et al. (2017). New fossils from Jebel Irhoud, Morocco and the Pan-African origin of Homo sapiens. Nature *546*, 289–292.

Huerta-Cepas, J., Forslund, K., Coelho, L.P., Szklarczyk, D., Jensen, L.J., von Mering, C., and Bork, P. (2017). Fast genome-wide functional annotation through orthology assignment by eggNOG-mapper. Mol. Biol. Evol. *34*, 2115–2122.

Huerta-Cepas, J., Szklarczyk, D., Forslund, K., Cook, H., Heller, D., Walter, M.C., Rattei, T., Mende, D.R., Sunagawa, S., Kuhn, M., et al. (2016). eggNOG 4.5: a hierarchical orthology framework with improved functional annotations for eukaryotic, prokaryotic and viral sequences. Nucleic Acids Res. *44*, D286–D293.

Human Microbiome Project Consortium (2012). Structure, function and diversity of the healthy human microbiome. Nature *486*, 207–214.

Hyatt, D., Chen, G.L., Locascio, P.F., Land, M.L., Larimer, F.W., and Hauser, L.J. (2010). Prodigal: prokaryotic gene recognition and translation initiation site identification. BMC Bioinformatics *11*, 119.

Jain, C., Rodriguez-R, L.M., Phillippy, A.M., Konstantinidis, K.T., and Aluru, S. (2018). High throughput ANI analysis of 90K prokaryotic genomes reveals clear species boundaries. Nat. Commun. *9*, 5114.

Janda, J.M., and Abbott, S.L. (2007). 16S rRNA gene sequencing for bacterial identification in the diagnostic laboratory: pluses, perils, and pitfalls. J. Clin. Microbiol. *45*, 2761–2764.

Jiménez, F.A., Gardner, S.L., Araújo, A., Fugassa, M., Brooks, R.H., Racz, E., and Reinhard, K.J. (2012). Zoonotic and human parasites of inhabitants of Cueva de los Muertos Chiquitos, Rio Zape Valley, Durango, Mexico. J. Parasitol. *98*, 304–309.

Jónsson, H., Ginolhac, A., Schubert, M., Johnson, P.L.F., and Orlando, L. (2013). mapDamage2.0: fast approximate Bayesian estimates of ancient DNA damage parameters. Bioinformatics *29*, 1682–1684.

Karlsson, F.H., Tremaroli, V., Nookaew, I., Bergström, G., Behre, C.J., Fagerberg, B., Nielsen, J., and Bäckhed, F. (2013). Gut metagenome in European women with normal, impaired and diabetic glucose control. Nature *498*, 99–103.

Katoh, K., and Standley, D.M. (2013). MAFFT multiple sequence alignment software version 7: improvements in performance and usability. Mol. Biol. Evol. *30*, 772–780.

Kaufman, L., and Rousseeuw, P.J. (1990). Finding groups in data. An introduction to cluster analysis.

Keller, A., Graefen, A., Ball, M., Matzas, M., Boisguerin, V., Maixner, F., Leidinger, P., Backes, C., Khairat, R., Forster, M., et al. (2012). New insights into the Tyrolean Iceman's origin and phenotype as inferred by whole-genome sequencing. Nat. Commun. *3*, 698.

Kircher, M., Sawyer, S., and Meyer, M. (2012). Double indexing overcomes inaccuracies in multiplex sequencing on the Illumina platform. Nucleic Acids Res. *40*, e3.

Konstantinidis, K.T., and Tiedje, J.M. (2005). Genomic insights that advance the species definition for prokaryotes. Proc. Natl. Acad. Sci. USA *102*, 2567–2572.

Korneliussen, T.S., Albrechtsen, A., and Nielsen, R. (2014). ANGSD: analysis of next generation sequencing data. BMC Bioinformatics *15*, 356.

Kostic, A.D., Gevers, D., Siljander, H., Vatanen, T., Hyötyläinen, T., Hämäläinen, A.-M., Peet, A., Tillmann, V., Pöhö, P., Mattila, I., et al. (2015). The dynamics of the human infant gut microbiome in development and in progression toward type 1 diabetes. Cell Host Microbe *17*, 260–273.

Kovatcheva-Datchary, P., Nilsson, A., Akrami, R., Lee, Y.S., De Vadder, F., Arora, T., Hallen, A., Martens, E., Björck, I., and Bäckhed, F. (2015). Dietary fiber-induced improvement in glucose metabolism is associated with increased abundance of Prevotella. Cell Metab. *22*, 971–982.

Langmead, B., and Salzberg, S.L. (2012). Fast gapped-read alignment with Bowtie 2. Nat. Methods *9*, 357–359.

Lazaridis, I., Nadel, D., Rollefson, G., Merrett, D.C., Rohland, N., Mallick, S., Fernandes, D., Novak, M., Gamarra, B., Sirak, K., et al. (2016). Genomic insights into the origin of farming in the ancient Near East. Nature *536*, 419–424.

Lazaridis, I., Patterson, N., Mittnik, A., Renaud, G., Mallick, S., Kirsanow, K., Sudmant, P.H., Schraiber, J.G., Castellano, S., Lipson, M., et al. (2014). Ancient human genomes suggest three ancestral populations for present-day Europeans. Nature *513*, 409–413.

Le Chatelier, E., Nielsen, T., Qin, J., Prifti, E., Hildebrand, F., Falony, G., Almeida, M., Arumugam, M., Batto, J.-M., Kennedy, S., et al. (2013). Richness of human gut microbiome correlates with metabolic markers. Nature *500*, 541–546.

Ley, R.E. (2016). Gut microbiota in 2015: Prevotella in the gut: choose carefully. Nat. Rev. Gastroenterol. Hepatol. *13*, 69–70.

Li, J., Jia, H., Cai, X., Zhong, H., Feng, Q., Sunagawa, S., Arumugam, M., Kultima, J.R., Prifti, E., Nielsen, T., et al. (2014). An integrated catalog of reference genes in the human gut microbiome. Nat. Biotechnol. *32*, 834–841.

Li, D., Liu, C.M., Luo, R., Sadakane, K., and Lam, T.W. (2015). MEGAHIT: an ultra-fast single-node solution for large and complex metagenomics assembly via succinct de Bruijn graph. Bioinformatics *31*, 1674–1676.

Li, H., Handsaker, B., Wysoker, A., Fennell, T., Ruan, J., Homer, N., Marth, G., Abecasis, G., and Durbin, R.; 1000 Genome Project Data Processing Subgroup (2009). The Sequence Alignment/Map format and SAMtools. Bioinformatics *25*, 2078–2079.

Li, J., Zhao, F., Wang, Y., Chen, J., Tao, J., Tian, G., Wu, S., Liu, W., Cui, Q., Geng, B., et al. (2017). Gut microbiota dysbiosis contributes to the development of hypertension. Microbiome *5*, 14.

Li, S.S., Zhu, A., Benes, V., Costea, P.I., Hercog, R., Hildebrand, F., Huerta-Cepas, J., Nieuwdorp, M., Salojärvi, J., and Voigt, A.Y. (2016). Durable coexistence of donor and recipient strains after fecal microbiota transplantation. Science *352*, 586–589.

Liu, W., Zhang, J., Wu, C., Cai, S., Huang, W., Chen, J., Xi, X., Liang, Z., Hou, Q., Zhou, B., et al. (2016). Unique features of Ethnic Mongolian Gut microbiome revealed by metagenomic analysis. Sci. Rep. *6*, 34826.

Llamas, B., Fehren-Schmitz, L., Valverde, G., Soubrier, J., Mallick, S., Rohland, N., Nordenfelt, S., Valdiosera, C., Richards, S.M., Rohrlach, A., et al. (2016). Ancient mitochondrial DNA provides high-resolution time scale of the peopling of the Americas. Sci. Adv. *2*, e1501385.

Loman, N.J., Constantinidou, C., Christner, M., Rohde, H., Chan, J.Z.-M., Quick, J., Weir, J.C., Quince, C., Smith, G.P., Betley, J.R., et al. (2013). A culture-independent sequence-based metagenomics approach to the investigation of an outbreak of Shiga-toxigenic Escherichia coli O104:H4. JAMA *309*, 1502–1510.

Lombard, V., Golaconda Ramulu, H., Drula, E., Coutinho, P.M., and Henrissat, B. (2014). The carbohydrate-active enzymes database (CAZy) in 2013. Nucleic Acids Res. *42*, D490–D495.

Louis, S., Tappu, R.-M., Damms-Machado, A., Huson, D.H., and Bischoff, S.C. (2016). Characterization of the Gut Microbial Community of Obese Patients Following a Weight-Loss Intervention Using Whole Metagenome Shotgun Sequencing. PLoS One *11*, e0149564.

Löytynoja, A. (2014). Phylogeny-aware alignment with PRANK. Methods Mol. Biol. *1079*, 155–170.

Lynnerup, N. (2007). Mummies. Am. J. Phys. Anthropol. *Suppl 45*, 162–190.

Maixner, F., Krause-Kyora, B., Turaev, D., Herbig, A., Hoopmann, M.R., Hallows, J.L., Kusebauch, U., Vigl, E.E., Malfertheiner, P., Megraud, F., et al. (2016). The 5300-year-old Helicobacter pylori genome of the Iceman. Science *351*, 162–165.

Maixner, F., Turaev, D., Cazenave-Gassiot, A., Janko, M., Krause-Kyora, B., Hoopmann, M.R., Kusebauch, U., Sartain, M., Guerriero, G., O'Sullivan, N., et al. (2018). The Iceman's last meal consisted of fat, wild meat, and cereals. Curr. Biol. *28*, 2348–2355.e9.

Meade, T. (1994). A dietary analysis of coprolites from a prehistoric Mexican cave site (University of Nebraska-Lincoln), Master's Thesis.

Meyer, M., and Kircher, M. (2010). Illumina sequencing library preparation for highly multiplexed target capture and sequencing. Cold Spring Harb. Protoc. *2010*, pdb.prot5448.

Morrow, J.J., and Reinhard, K.J. (2016). Cryptosporidium parvum among coprolites from la Cueva de los muertos Chiquitos (600–800 CE), Rio zape Valley, Durango, Mexico. J. Parasitol. *102*, 429–435.

Müller, W., Fricke, H., Halliday, A.N., McCulloch, M.T., and Wartho, J.A. (2003). Origin and migration of the Alpine Iceman. Science *302*, 862–866.

Nielsen, H.B., Almeida, M., Juncker, A.S., Rasmussen, S., Li, J., Sunagawa, S., Plichta, D.R., Gautier, L., Pedersen, A.G., Le Chatelier, E., et al. (2014). Identification and assembly of genomes and genetic elements in complex metagenomic samples without using reference genomes. Nat. Biotechnol. *32*, 822–828.

Nurk, S., Meleshko, D., Korobeynikov, A., and Pevzner, P.A. (2017). metaSPAdes: a new versatile metagenomic assembler. Genome Res. *27*, 824–834.

Obregon-Tito, A.J., Tito, R.Y., Metcalf, J., Sankaranarayanan, K., Clemente, J.C., Ursell, L.K., Zech Xu, Z., Van Treuren, W., Knight, R., Gaffney, P.M., et al. (2015). Subsistence strategies in traditional societies distinguish gut microbiomes. Nat. Commun. *6*, 6505.

Orlando, L., Gilbert, M.T.P., and Willerslev, E. (2015). Reconstructing ancient genomes and epigenomes. Nat. Rev. Genet. *16*, 395–408.

Page, A.J., Cummins, C.A., Hunt, M., Wong, V.K., Reuter, S., Holden, M.T.G., Fookes, M., Falush, D., Keane, J.A., and Parkhill, J. (2015). Roary: rapid large-scale prokaryote pan genome analysis. Bioinformatics *31*, 3691–3693.

Parks, D.H., Imelfort, M., Skennerton, C.T., Hugenholtz, P., and Tyson, G.W. (2015). CheckM: assessing the quality of microbial genomes recovered from isolates, single cells, and metagenomes. Genome Res. *25*, 1043–1055.

Pasolli, E., Asnicar, F., Manara, S., Zolfo, M., Karcher, N., Armanini, F., Beghini, F., Manghi, P., Tett, A., Ghensi, P., et al. (2019). Extensive unexplored human microbiome diversity revealed by over 150,000 genomes from metagenomes spanning age, geography, and lifestyle. Cell *176*, 649–662.e20.

Pasolli, E., Schiffer, L., Manghi, P., Renson, A., Obenchain, V., Truong, D.T., Beghini, F., Malik, F., Ramos, M., Dowd, J.B., et al. (2017). Accessible, curated metagenomic data through ExperimentHub. Nat. Methods *14*, 1023–1024.

Patterson, N., Moorjani, P., Luo, Y., Mallick, S., Rohland, N., Zhan, Y., Genschoreck, T., Webster, T., and Reich, D. (2012). Ancient admixture in human history. Genetics *192*, 1065–1093.

Patterson, N., Price, A.L., and Reich, D. (2006). Population structure and eigenanalysis. PLoS Genet. *2*, e190.

Pedersen, H.K., Gudmundsdottir, V., Nielsen, H.B., Hyotylainen, T., Nielsen, T., Jensen, B.A.H., Forslund, K., Hildebrand, F., Prifti, E., Falony, G., et al. (2016). Human gut microbes impact host serum metabolome and insulin sensitivity. Nature *535*, 376–381.

Perego, U.A., Angerhofer, N., Pala, M., Olivieri, A., Lancioni, H., Hooshiar Kashani, B., Carossa, V., Ekins, J.E., Gómez-Carballa, A., Huber, G., et al. (2010). The initial peopling of the Americas: a growing number of founding mitochondrial genomes from Beringia. Genome Res. *20*, 1174–1179.

Posth, C., Nakatsuka, N., Lazaridis, I., Skoglund, P., Mallick, S., Lamnidis, T.C., Rohland, N., Nägele, K., Adamski, N., Bertolini, E., et al. (2018). Reconstructing the deep population history of Central and South America. Cell *175*, 1185–1197.e22.

Price, A.L., Patterson, N.J., Plenge, R.M., Weinblatt, M.E., Shadick, N.A., and Reich, D. (2006). Principal components analysis corrects for stratification in genome-wide association studies. Nat. Genet. *38*, 904–909.

Price, M.N., Dehal, P.S., and Arkin, A.P. (2010). FastTree 2–approximately maximum-likelihood trees for large alignments. PLoS One *5*, e9490.

Pritchard, L., Glover, R.H., Humphris, S., Elphinstone, J.G., and Toth, I.K. (2015). Genomics and taxonomy in diagnostics for food security: soft-rotting enterobacterial plant pathogens. Anal. Methods *8*, 12–24.

Qin, J., Li, R., Raes, J., Arumugam, M., Burgdorf, K.S., Manichanh, C., Nielsen, T., Pons, N., Levenez, F., Yamada, T., et al. (2010). A human gut microbial gene catalogue established by metagenomic sequencing. Nature *464*, 59–65.

Qin, J., Li, Y., Cai, Z., Li, S., Zhu, J., Zhang, F., Liang, S., Zhang, W., Guan, Y., Shen, D., et al. (2012). A metagenome-wide association study of gut microbiota in type 2 diabetes. Nature *490*, 55–60.

Qin, N., Yang, F., Li, A., Prifti, E., Chen, Y., Shao, L., Guo, J., Le Chatelier, E., Yao, J., Wu, L., et al. (2014). Alterations of the human gut microbiome in liver cirrhosis. Nature *513*, 59–64.

Rambaut, A., Drummond, A.J., Xie, D., Baele, G., and Suchard, M.A. (2018). Posterior summarization in bayesian phylogenetics using Tracer 1.7. Syst. Biol. *67*, 901–904.

Rampelli, S., Schnorr, S.L., Consolandi, C., Turroni, S., Severgnini, M., Peano, C., Brigidi, P., Crittenden, A.N., Henry, A.G., and Candela, M. (2015). Metagenome sequencing of the Hadza hunter-gatherer gut microbiota. Curr. Biol. *25*, 1682–1693.

Raymond, F., Ouameur, A.A., Déraspe, M., Iqbal, N., Gingras, H., Dridi, B., Leprohon, P., Plante, P.-L., Giroux, R., Bérubé, È., et al. (2016). The initial state of the human gut microbiome determines its reshaping by antibiotics. ISME J. *10*, 707–720.

Renaud, G., Slon, V., Duggan, A.T., and Kelso, J. (2015). Schmutzi: estimation of contamination and endogenous mitochondrial consensus calling for ancient DNA. Genome Biol. *16*, 224.

Rosenbloom, K.R., Armstrong, J., Barber, G.P., Casper, J., Clawson, H., Diekhans, M., Dreszer, T.R., Fujita, P.A., Guruvadoo, L., Haeussler, M., et al. (2015). The UCSC Genome Browser database: 2015 update. Nucleic Acids Res. *43*, D670–D681.

Scher, J.U., Sczesnak, A., Longman, R.S., Segata, N., Ubeda, C., Bielski, C., Rostron, T., Cerundolo, V., Pamer, E.G., Abramson, S.B., et al. (2013). Expansion of intestinal Prevotella copri correlates with enhanced susceptibility to arthritis. ELife *2*, e01202.

Schirmer, M., Smeekens, S.P., Vlamakis, H., Jaeger, M., Oosting, M., Franzosa, E.A., Ter Horst, R., Jansen, T., Jacobs, L., Bonder, M.J., et al. (2016). Linking the Human Gut Microbiome to Inflammatory Cytokine Production Capacity. Cell *167*, 1125–1136.e8.

Schnorr, S.L., Candela, M., Rampelli, S., Centanni, M., Consolandi, C., Basaglia, G., Turroni, S., Biagi, E., Peano, C., Severgnini, M., et al. (2014). Gut microbiome of the Hadza hunter-gatherers. Nat. Commun. *5*, 3654.

Scholz, M., Ward, D.V., Pasolli, E., Tolio, T., Zolfo, M., Asnicar, F., Truong, D.T., Tett, A., Morrow, A.L., and Segata, N. (2016). Strain-level microbial epidemiology and population genomics from shotgun metagenomics. Nat. Methods *13*, 435–438.

Seemann, T. (2014). Prokka: rapid prokaryotic genome annotation. Bioinformatics *30*, 2068–2069.

Segata, N. (2015). Gut microbiome: westernization and the disappearance of intestinal diversity. Curr. Biol. *25*, R611–R613.

Segata, N., Börnigen, D., Morgan, X.C., and Huttenhower, C. (2013). PhyloPhlAn is a new method for improved phylogenetic and taxonomic placement of microbes. Nat. Commun. *4*, 2304.

Skoglund, P., Storå, J., Götherström, A., and Jakobsson, M. (2013). Accurate sex identification of ancient human remains using DNA shotgun sequencing. J. Archaeol. Sci. *40*, 4477–4482.

Smits, S.A., Leach, J., Sonnenburg, E.D., Gonzalez, C.G., Lichtman, J.S., Reid, G., Knight, R., Manjurano, A., Changalucha, J., Elias, J.E., et al. (2017). Seasonal cycling in the gut microbiome of the Hadza hunter-gatherers of Tanzania. Science *357*, 802–806.

Sonnenburg, E.D., Smits, S.A., Tikhonov, M., Higginbottom, S.K., Wingreen, N.S., and Sonnenburg, J.L. (2016). Diet-induced extinctions in the gut microbiota compound over generations. Nature *529*, 212–215.

Sonnenburg, J.L., and Bäckhed, F. (2016). Diet-microbiota interactions as moderators of human metabolism. Nature *535*, 56–64.

Spindler, K. (1994). The man in the ice (Weidenfeld & Nicolson).

Stamatakis, A. (2014). RAxML version 8: a tool for phylogenetic analysis and post-analysis of large phylogenies. Bioinformatics *30*, 1312–1313.

Statovci, D., Aguilera, M., MacSharry, J., and Melgar, S. (2017). The impact of Western diet and nutrients on the microbiota and immune response at mucosal interfaces. Front. Immunol. *8*, 838.

Tackney, J.C., Potter, B.A., Raff, J., Powers, M., Watkins, W.S., Warner, D., Reuther, J.D., Irish, J.D., and O'Rourke, D.H. (2015). Two contemporaneous mitogenomes from terminal Pleistocene burials in eastern Beringia. Proc. Natl. Acad. Sci. USA *112*, 13833–13838.

Tamm, E., Kivisild, T., Reidla, M., Metspalu, M., Smith, D.G., Mulligan, C.J., Bravi, C.M., Rickards, O., Martinez-Labarga, C., Khusnutdinova, E.K., et al. (2007). Beringian standstill and spread of Native American founders. PLoS One *2*, e829.

Tang, J.N., Zeng, Z.G., Wang, H.N., Yang, T., Zhang, P.J., Li, Y.L., Zhang, A.Y., Fan, W.Q., Zhang, Y., Yang, X., et al. (2008). An effective method for isolation of DNA from pig faeces and comparison of five different methods. J. Microbiol. Methods *75*, 432–436.

Thomas, A.M., Manghi, P., Asnicar, F., Pasolli, E., Armanini, F., Zolfo, M., Beghini, F., Manara, S., Karcher, N., Pozzi, C., et al. (2019). Metagenomic analysis of colorectal cancer datasets identifies cross-cohort microbial diagnostic signatures and a link with choline degradation. Nat. Med. *25*, 667–678.

Tibshirani, R., and Walther, G. (2005). Cluster validation by prediction strength. J. Comput. Graph. Stat. *14*, 511–528.

Tito, R.Y., Knights, D., Metcalf, J., Obregon-Tito, A.J., Cleeland, L., Najar, F., Roe, B., Reinhard, K., Sobolik, K., Belknap, S., et al. (2012). Insights from characterizing extinct human gut microbiomes. PLoS One *7*, e51146.

Tito, R.Y., Macmil, S., Wiley, G., Najar, F., Cleeland, L., Qu, C., Wang, P., Romagne, F., Leonard, S., Ruiz, A.J., et al. (2008). Phylotyping and functional analysis of two ancient human microbiomes. PLoS One *3*, e3703.

Truong, D.T., Franzosa, E.A., Tickle, T.L., Scholz, M., Weingart, G., Pasolli, E., Tett, A., Huttenhower, C., and Segata, N. (2015). MetaPhlAn2 for enhanced metagenomic taxonomic profiling. Nat. Methods *12*, 902–903.

Truong, D.T., Tett, A., Pasolli, E., Huttenhower, C., and Segata, N. (2017). Microbial strain-level population structure and genetic diversity from metagenomes. Genome Res. *27*, 626–638.

Ungar, P.S., and Sponheimer, M. (2011). The diets of early hominins. Science *334*, 190–193.

van der Walt, A.J., van Goethem, M.W., Ramond, J.B., Makhalanyane, T.P., Reva, O., and Cowan, D.A. (2017). Assembling metagenomes, one community at a time. BMC Genomics *18*, 521.

Vangay, P., Johnson, A.J., Ward, T.L., Al-Ghalith, G.A., Shields-Cutler, R.R., Hillmann, B.M., Lucas, S.K., Beura, L.K., Thompson, E.A., Till, L.M., et al.

(2018). US immigration westernizes the human gut microbiome. Cell *175*, 962–972.e10.

Vatanen, T., Kostic, A.D., d'Hennezel, E., Siljander, H., Franzosa, E.A., Yassour, M., Kolde, R., Vlamakis, H., Arthur, T.D., Hämäläinen, A.-M., et al. (2016). Variation in Microbiome LPS Immunogenicity Contributes to Autoimmunity in Humans. Cell *165*, 1551.

Vincent, C., Miller, M.A., Edens, T.J., Mehrotra, S., Dewar, K., and Manges, A.R. (2016). Bloom and bust: intestinal microbiota dynamics in response to hospital exposures and Clostridium difficile colonization or infection. Microbiome *4*, https://doi.org/10.1186/s40168-016-0156-3.

Vogtmann, E., Hua, X., Zeller, G., Sunagawa, S., Voigt, A.Y., Hercog, R., Goedert, J.J., Shi, J., Bork, P., and Sinha, R. (2016). Colorectal cancer and the human gut microbiome: reproducibility with whole-genome shotgun sequencing. PLoS One *11*, e0155362.

Weissensteiner, H., Pacher, D., Kloss-Brandstätter, A., Forer, L., Specht, G., Bandelt, H.J., Kronenberg, F., Salas, A., and Schönherr, S. (2016). HaploGrep 2: Mitochondrial haplogroup classification in the era of high-throughput sequencing. Nucleic Acids Res. *44*, W58–W63.

Wen, C., Zheng, Z., Shao, T., Liu, L., Xie, Z., Le Chatelier, E., He, Z., Zhong, W., Fan, Y., Zhang, L., et al. (2017). Quantitative metagenomics reveals unique gut microbiome biomarkers in ankylosing spondylitis. Genome Biol. *18*, 142.

Wood, B., and Collard, M. (1999). The human genus. Science *284*, 65–71.

Xie, H., Guo, R., Zhong, H., Feng, Q., Lan, Z., Qin, B., Ward, K.J., Jackson, M.A., Xia, Y., Chen, X., et al. (2016). Shotgun Metagenomics of 250 Adult Twins Reveals Genetic and Environmental Impacts on the Gut Microbiome. Cell Syst. *3*, 572–584.e3.

Yatsunenko, T., Rey, F.E., Manary, M.J., Trehan, I., Dominguez-Bello, M.G., Contreras, M., Magris, M., Hidalgo, G., Baldassano, R.N., Anokhin, A.P., et al. (2012). Human gut microbiome viewed across age and geography. Nature *486*, 222–227.

Yin, Y., Mao, X., Yang, J., Chen, X., Mao, F., and Xu, Y. (2012). dbCAN: a web resource for automated carbohydrate-active enzyme annotation. Nucleic Acids Res. *40*, W445–W451.

Yu, J., Feng, Q., Wong, S.H., Zhang, D., Liang, Q.Y., Qin, Y., Tang, L., Zhao, H., Stenvang, J., Li, Y., et al. (2017). Metagenomic analysis of faecal microbiome as a tool towards targeted non-invasive biomarkers for colorectal cancer. Gut *66*, 70–78.

Zeevi, D., Korem, T., Zmora, N., Israeli, D., Rothschild, D., Weinberger, A., Ben-Yacov, O., Lador, D., Avnit-Sagi, T., Lotan-Pompan, M., et al. (2015). Personalized Nutrition by Prediction of Glycemic Responses. Cell *163*, 1079–1094.

Zeller, G., Tap, J., Voigt, A.Y., Sunagawa, S., Kultima, J.R., Costea, P.I., Amiot, A., Böhm, J., Brunetti, F., Habermann, N., et al. (2014). Potential of fecal microbiota for early-stage detection of colorectal cancer. Mol. Syst. Biol. *10*, 766.

STAR★METHODS

KEY RESOURCES TABLE

REAGENT or RESOURCE	SOURCE	IDENTIFIER
Biological Samples		
Faecal samples from a Ethiopian cohort	This study	N/A
Faecal samples from a Ghanaian cohort	This study	N/A
Faecal samples from a Tanzanian cohort	This study	N/A
Critical Commercial Assays		
PowerSoil DNA isolation kit	Qiagen	Cat No./ID: 12888-100
TruSeq DNA PCR-free Library Prep Kit	Illumina, California, USA	20015962
NexteraXT DNA Library Preparation Kit	Illumina, California, USA	FC-131-1096
Deposited Data		
Raw sequencing data (Ethiopia)	Pasolli et al., 2019 and this study	NCBI-SRA BioProject: PRJNA504891
Raw sequencing data (Ghana)	This study	NCBI-SRA BioProject: PRJNA529124
Raw sequencing data (Tanzania)	This study	NCBI-SRA BioProject: PRJNA529400
All *P. copri* isolate genomes and MAGs	This study	http://segatalab.cibio.unitn.it/data/Pcopri_Tett_et_al.html
Raw sequencing data (Ancient metagenomics samples)	This study	NCBI-SRA BioProject: PRJEB31971
Software and Algorithms		
metaSPAdes (version 3.10.1)	Nurk et al., 2017	https://github.com/ablab/spades/releases
MEGAHIT (version 1.1.1)	Li et al., 2015	https://github.com/voutcn/megahit
anvi'o (version 2.3.2)	Eren et al., 2015	https://github.com/merenlab/anvio
MetaPhlAn2 (version 2.0)	Truong et al., 2015	https://bitbucket.org/biobakery/metaphlan2
Bowtie2 (version 2.2.9)	Langmead and Salzberg, 2012	https://github.com/BenLangmead/bowtie2
blastn (version 2.6.0+)	Altschul et al., 1990	ftp://ftp.ncbi.nlm.nih.gov/blast/executables/blast
CMseq (version dev commit 41082ef)		https://bitbucket.org/CibioCM/cmseq/
Prokka (version 1.11)	Seemann, 2014	https://github.com/tseemann/prokka
Pyani (version 0.2.6)	Pritchard et al., 2015	https://github.com/widdowquinn/pyani
Roary (version 3.11)	Page et al., 2015	https://github.com/sanger-pathogens/Roary
SciPy	Bressert, 2012	https://github.com/scipy/scipy
PhyloPhlAn (version dev commit 7c38e19)	Segata et al., 2013	https://bitbucket.org/nsegata/phylophlan
Diamond (version v0.9.9.110)	Buchfink et al., 2015	https://github.com/bbuchfink/diamond
MAFFT (version v7.310)	Katoh and Standley, 2013	https://github.com/The-Bioinformatics-Group/Albiorix/wiki/mafft
trimAl (version 1.2rev59)	Capella-Gutiérrez et al., 2009	https://github.com/scapella/trimal
FastTree (version 2.1.10)	Price et al., 2010	https://github.com/PavelTorgashov/FastTree
RAxML (version 8.1.15)	Stamatakis, 2014	https://github.com/stamatak/standard-RAxML
GraPhlAn (version 1.1.3)	Asnicar et al., 2015	https://bitbucket.org/nsegata/graphlan/
EggNOG mapper (version 1.0.3)	Huerta-Cepas et al., 2017	https://github.com/jhcepas/eggnog-mapper
HMMSEARCH (version 3.1b2)	Eddy, 2011	https://github.com/guyz/HMM
DeDup		https://github.com/apeltzer/DeDup
mapDamage2	Jónsson et al., 2013	https://github.com/ginolhac/mapDamage
SAMtools	Li et al., 2009	https://github.com/samtools
HaploGrep2	Weissensteiner et al., 2016	https://github.com/seppinho/haplogrep-cmd
PileupCaller		https://github.com/stschiff/sequenceTools/tree/master/src-pileupCaller
BEAST (version 2.5.1)	Bouckaert et al., 2014	https://github.com/CompEvol/beast2
Tracer (version 1.7)	Rambaut et al., 2018	https://github.com/beast-dev/tracer/

(*Continued on next page*)

Continued

REAGENT or RESOURCE	SOURCE	IDENTIFIER
Schmutzi	Renaud et al., 2015	https://github.com/grenaud/schmutzi
ANGSD	Korneliussen et al., 2014	https://github.com/ANGSD/angsd
PRANK (version 140603)	Löytynoja, 2014	https://github.com/ariloytynoja/prank-msa
Prodigal (version 2.6.3)		https://github.com/hyattpd/Prodigal
Barrnap (version 0.9)		https://github.com/tseemann/barrnap

LEAD CONTACT AND MATERIALS AVAILABILITY

Further information and requests for resources and reagents should be directed to and will be fulfilled by the Lead Contact, Nicola Segata (nicola.segata@unitn.it). This study did not generate new unique reagents.

EXPERIMENTAL MODEL AND SUBJECT DETAILS

All subjects enrolled in this study included infants and adults from Ghana, Tanzania and Ethiopia as described in the STAR Methods. All individuals sampled in this study were healthy defined as free of self-reported disease. For Ghana, 44 individuals were included (23 adults, avg age (yrs): 33.14 (s.d.: 6.61), range: 20-45, sex: 52.2% female, 47.8% male; 25 children, avg age (yrs): 6.81 (s.d. 3.67), range 0-15 yrs). For Ethiopia 50 individuals (24 adults, avg age (yrs): 29.35 (s.d. 6.24), range: 22-45, sex: 100% female; 26 infants, avg age (yrs): 1.31 (s.d.1.05), range 0.167-4.5 yrs). And for Tanzania 68 individuals (37 adults, avg age (yrs): 29.27 (s.d. 8.88), range: 18-61, sex: 48.65% female, 51.35% male; 31 children, avg age (yrs): 8.22 (s.d. 4.74), range 0-18 yrs). Ethical approval for the Ethiopian Cohort was granted by the by the Research Ethics Committee of the Valencia University (reference number: H1484811493170) and by the Ethics Committee of the Consejo Superior de Investigaciones Cientìficas (Madrid, Spain), number 058/2018. For the Ghanian and Tanzanian cohorts ethical approval was granted by the Ethics Committee of the Ärztekammer Hamburg (Germany; PV5075), the Committee On Human Research, Publications And Ethics, Kwame Nkrumah University Of Science And Technology (Ghana; CHRPE/AP/440/18) and the Medical Research Coordinating Committee of the National Institute for Medical Research (Tanzania; NIMR/HQ/R.8a/Vol. IX/2252).

METHOD DETAILS

The overall aim of the study was to identify and reconstruct *P. copri* genomes from publicly available intestinal metagenomic datasets including non-Westernized datasets. These datasets represent multiple countries, host-conditions and lifestyles. The process involved collating a panel of 72 high-quality *P. copri* genomes comprising manually curated metagenome assembled genomes, the *P. copri* isolates sequenced in this study and publicly available reference sequences. The resulting panel of genomes was used to automatically reconstruct additional *P. copri* genomes from single-sample assembled metagenomes. In addition, clade-specific markers were designed to accurately identify the presence and abundance of the *P. copri* clades across datasets.

Metagenomic Assembly

Metagenomic samples were assembled using metaSpades (version 3.10.1) (Nurk et al., 2017) using default parameters, chosen due to its reported performance compared to other assemblers (Forouzan et al., 2018; Pasolli et al., 2019; van der Walt et al., 2017). Samples that exceeded permitted memory requirements (>1Tb of RAM) or those for which only unpaired reads were available were assembled using Megahit (version 1.1.1) (Li et al., 2015) using default parameters. Only assembled contigs $\geq$ 1kb were considered further.

Constructing a *P. copri* Genome Panel with Additional Sequenced Isolates and Manually Curated Genomes from Metagenomes

A panel of 72 *P. copri* genomes were collated consisting of two publicly available reference genomes (RefSeq assembly accessions: GCF_000157935.1, GCF_002224675.1), 15 isolate genomes sequenced in this study (see below) and 55 manually curated metagenome assembled genomes.

P. copri strains were isolated from stool from healthy subjects and new onset rheumatoid arthritis patients. Stool was collected into anaerobic transport media (Anaerobe Systems), then streaked on BRU and LKV plates (Anaerobe Systems). After 24-48h, individual colonies were picked and screened with *Prevotella*-specific PCR primers, and the 16S rRNA V3-V4 sequence was confirmed by Sanger sequencing (Fehlner-Peach et al., 2019). *Prevotella*-positive isolates were grown on BRU plates, and mature colonies were collected for genomic DNA isolation with the PowerSoil DNA isolation kit (Qiagen). Libraries were prepared for sequencing

on the HiSeq2500 platform with the TruSeq DNA PCR-free Library Prep Kit (Illumina). In total 83 *P. copri* isolates were sequenced. As this included multi-sampling from the same individual, 15 isolates were selected for this study that represented the total genetic diversity of the isolate dataset.

The 55 genome bins were reconstructed using anvi'o (version 2.3.2) (Eren et al., 2015) applied on a set of assembled metagenomes. Anvi'o provides a platform for metagenomic genome binning and offers the ability to manually asses and curate those bins, potentially increasing accuracy compared to automated binning methods, but at the expense of being low throughput. Briefly, the 100 metagenomic samples determined to have a high abundance of *P. copri* based on MetaPhlAn2 (Truong et al., 2015) were selected. The metagenomic samples were assembled (see above) and reads mapped back to the contigs using Bowtie2 (version 2.2.9, using "very-sensitive-local" parameter) (Langmead and Salzberg, 2012). Contigs (>2.5kb) were clustered by anvi'o based on coverage and tetranucleotide frequency, and manually curated. All reconstructed bins were subjected to strict quality control (see below), resulting in 55 high-quality genome bins.

Automated Reconstruction of *P. copri* Genomes from >6500 Metagenomes

To automatically reconstructed *P.copri* genomes from metagenomes, we first assembled each metagenome (see above) and for each assembly its contigs were mapped against the panel of 72 high-quality reference genomes representing the four clades of the *P. copri* complex (described above) using Blastn (version 2.6.0+) (Altschul et al., 1990). Only contigs with a nucleotide identity ≥95% and an alignment ≥50% were considered further and placed into one of the four *P. copri* bins (Clade A, B, C or D) based on the membership of the reference genome. On the rare occasion a contig was ≥95% identical and aligned over ≥50% to multiple reference genomes representing different clades, the contig was placed into a single clade bin based on best BitScore, if this score was ≥10% than any other competing clade(s). If the BitScore threshold was not satisfied, the contig could not confidently be placed and was not considered further. All reconstructed *P. copri* metagenomic genomes were assessed for quality (see below).

P. copri Genome Quality Control

All *P. copri* genomes were strictly quality controlled. QC involved four steps 1) genome size 2) estimated completeness, 3) estimated contamination and 4) level of strain heterogeneity. Only genome bins >2.5 Mb <5.0 Mb and composed of <500 contigs were considered. CheckM (Parks et al., 2015) was used to estimate the completeness and level of contamination. High quality genomes were those >95% completeness, <5% contamination, except for *P. copri* clade D where a completeness of >90% was used to be more inclusive. For the recently sequenced non-Westernized datasets (see section: Westernisation and additional Non-Westernized datasets, below) and the manually curated metagenomically assembled genomes a threshold of >90% completeness was also selected. We also investigated strain-level diversity for each of the *P. copri* clades within a sample as this could indicate contig chimeric assembly. Strain-level heterogeneity was estimated using an in-house developed tool, CMseq, available here: https://bitbucket.org/CibioCM/cmseq/commits/41082ef. Firstly, protein coding genes of the contigs were predicted with prodigal (version 2.6.3) (Hyatt et al., 2010) implemented in the Prokka pipeline (version 1.11) (Seemann, 2014). To avoid overestimating strain-heterogeneity due to genes in common across the four *P. copri* clades, only the clade specific genes (see below) were considered as a proxy to estimate strain heterogeneity. Secondly, metagenomic reads were mapped to the assembled contigs using Bowtie2 (Langmead and Salzberg, 2012) (version 2.2.9, using "very-sensitive-local" parameter) and for each coding nucleotide base calls were only considered if there was >10X coverage and a PHRED quality score of ≥ 30. Each position was considered non-polymorphic if the frequency of the dominant allele was >80%. When calculating the overall contig polymorphic rate only the non-synonymous positions were considered.

Genetic Distance between and within the *P. copri* Complex and Related Species

The average nucleotide distance (ANI) pairwise distances were calculated using pyani (version 0.2.6; option '-m ANIb') (Pritchard et al., 2015) for a subset of *P. copri* genomes representing the four clades (25 for clade A, B and C, and all the 15 genomes of clade D) and publicly available reference genomes of the *Prevotella*, *Alloprevotella* and *Paraprevotella* genera available from NCBI RefSeq. Distances scores were filtered to include only pairwise comparison where alignment lengths exceeded 500,000 bp. Pairwise core genome distances based on comparisons of single nucleotide polymorphisms were calculated on the core genome alignment of all 1,023 *P. copri* genomes. The core genome alignments were produced utilizing PRANK (Löytynoja, 2014) as part of the Roary pipeline (version 3.11) (Page et al., 2015) with the parameters of 90% similarity identity for gene clustering and present in 90% of genomes for defining core genes. The pangenome-based matrix also produced from Roary was used to compare the pairwise gene content similarity calculated using the Jaccard similarity coefficient as part of the SciPy package (Bressert, 2012). To infer instances of strain sharing between individuals, normalised phylogenetic distances on the *P. copri* phylogeny were compared and called as the same strain based on a 0.2% identity threshold as previously described (Truong et al., 2017).

Phylogenetic Analysis

The phylogenetic analyses were performed with PhyloPhlAn (Segata et al., 2013) using the new version available in the "dev" branch of the repository (commit 7c38e19, https://bitbucket.org/nsegata/phylophlan).

The phylogeny in Figure 1A was built using the 400 universal marker genes as identified by PhyloPhlAn using the following parameters: "–diversity low –fast". The set of external tools with their respective options is reported below:

- Diamond version v0.9.9.110, (Buchfink et al., 2015), with "Blastx" for the nucleotide-based mapping, "Blastp" for the amino-acid based mapping, and "–more-sensitive –id 50 –max-hsps 35 -k 0" in both cases
- MAFFT version v7.310, (Katoh and Standley, 2013), with "–localpair –maxiterate 1000 –anysymbol –auto" options
- trimAl version 1.2rev59, (Capella-Gutiérrez et al., 2009), with "-gappyout" option
- FastTree version 2.1.10, (Price et al., 2010), with "-mlacc 2 -slownni -spr 4 -fastest -mlnni 4 -no2nd -gtr -nt" options
- RAxML version 8.1.15 (Stamatakis, 2014), with "-p 1989 -m GTRCAT -t <FastTree phylogeny>" options

The tree was built on a total of 90 genomes composing a subset of 25 representative genomes for the three clades A, B and C whereas for clade D all 15 genomes were considered.

The phylogeny in http://segatalab.cibio.unitn.it/data/Pcopri_Tett_et_al.html and Figure S1B is based on the 210 set of core genes screened to be monophyletic as described in the section below on molecular dating. The phylogeny has been reconstructed using PhyloPhlAn with the following parameters: "–diversity low –trim greedy –remove_fragmentary_entries". Additionally, the set of external tools with their options is reported below:

- Blastn version 2.6.0+, (Altschul et al., 1990), with "-outfmt 6 -max_target_seqs 1000000" options
- MAFFT, trimAl, FastTree, and RAxML were run with the same options as reported above.

The phylogenies in Figure 2 and in Figure S3 were built with PhyloPhlAn using the set of core genes for each *P. copri* clade (>95% shared across all genomes within a clade) determined using Roary (version 3.11) (Page et al., 2015) with a minimum gene identity of 90%. PhyloPhlAn was run using the following parameters: "–mutation_rates –min_num_entries <97% of the number of input genomes> –diversity low". The set of external tools used is: Blastn, MAFFT, trimAl, FastTree, and RAxML, and they were executed with the same options as reported above. The phylogenetic trees in Figure 2, and Figure S3 were visualized using GraPhlAn (version 1.1.3) (Asnicar et al., 2015).

The *P. copri* Pangenome and Evaluating Prevalence and Abundance

The protein coding regions for the 72 *P. copri* genome panel (see above) were predicted using Prodigal (Hyatt et al., 2010) as part of the prokka pipeline (version 1.11) (Seemann, 2014) and the total *P. copri* pangenome determined using Roary with 90% similarity identity parameter (version 3.11) (Page et al., 2015). Markers specific to each clade of the *P. copri* complex where defined as present in >95% of the *P. copri* genomes of a given clade but absent in all others. This gave for Clade A n=430 markers, for Clade B n=954, for Clade C n=479 and for Clade D n=585. To determine if a *P. copri* clade is present in a metagenomic sample reads were mapped to the clade specific markers using Bowtie2 (Langmead and Salzberg, 2012) and mappings processed using PanPhlAn (Scholz et al., 2016). A marker was scored present if had a coverage $\geq$0.5X, and a clade present if $\geq$50% of the clade specific markers were hit. Estimation of *P. copri* clade relative abundance was calculated thus: (Mean clade marker coverage x approximated genome size (bp)) / total metagenome size (bp).

Westernisation and Additional Non-Westernized Datasets

Westernisation as the adoption of a Westernized lifestyle and culture can trace its origins to industrialisation and its promotion of urbanisation over the past two centuries. Westernisation has had a profound effect on human populations, due to access to health-care and pharmaceutical products, hygiene and sanitation, changes in diet (typically processed, high-fat, low in complex carbohydrates but rich in refined sugars and salt), population density increase and reduced exposure to livestock. Westernisation is nonetheless difficult to ascribe as it demarcates populations which are clearly on a continuum. For example certain non-Westernized populations such as Inuits typically consume a diet high in fat. In this study the definition of "Westernized" and "non-Westernized" is considered based on how populations differ on the above criteria and how the samples were reported in the original publication.

In this study five previous datasets were considered where non-Westernized populations have been sampled from Fiji (Brito et al., 2016), Peru (Obregon-Tito et al., 2015), Tanzania (Rampelli et al., 2015; Smits et al., 2017) and Mongolia (Liu et al., 2016). In addition, we recently sequenced a population of adults from a rainforest region in North-eastern Madagascar (110 metagenomes) (Pasolli et al., 2019). We expanded upon a dataset of 5 samples sequenced from an established cohort in Ethiopia from Gimbichu in the Oromia region (Pasolli et al., 2019) with 45 additional samples. This cohort included 24 mothers and their infant(s) for a total of 50 metagenomes. We also sequenced two non-Westernized populations from Ghana and Tanzania. In Ghana 12 extended families from the Asante Akim North district region were sampled where the local occupation is subsistence farming and the wider economy based on farming cash crops such as cocoa and plantain and there is also a commercial poultry industry (44 metagenomes). From Tanzania samples were collected from 18 families from Korogwe District region where local employment and economy is based on agriculture particularly based on sisal fibres, cashew nuts and cotton (68 metagenomes). For all samples DNA was extracted using the PowerSoil DNA isolation kit (Qiagen) as previously described (Human Microbiome Project Consortium, 2012). Libraries were constructed using the NexteraXT DNA Library Preparation Kit (Illumina) and sequenced on the Illumina HiSeq2500 100nt paired end platform with a target depth of 5Gb/sample.

Genome Functional Potential Analysis

We performed the functional annotation using the EggNOG mapper (version 1.0.3) (Huerta-Cepas et al., 2017) that is based on the EggNOG orthology system (Huerta-Cepas et al., 2016) and the sequence searches performed using HMMER (Eddy, 2011). We used

the KEGG Brite Hierarchy to screen the EggNOG annotations that are shown in Figure 4A. In this figure, for each clade, we report only the eggNOGs that are significantly different in each of its three pairwise comparisons to the other clades (p-value < 0.01, Bonferroni corrected Fisher-exact test). CAZy enzymes (Lombard et al., 2014) (http://www.cazy.org/) were predicted with HMMSEARCH (version 3.1b2) (Eddy, 2011) against dbCAN HMMs v6 using default parameters and applying post-processing stringency cut-offs as suggested (Yin et al., 2012). Figure 4C all the CAZy families that are significantly different (whether enriched or depleted) in at least one clade with respect to each of the other three clades considered separately (i.e. significant based on pairwise comparisons, Bonferroni corrected Fisher-exact test).

Inferring *P. copri* Sub-clades Based on Function

Sub-clades used in Figure S2B were inferred from the EggNOG functional profiles for each *P. copri* genome (above). The genomes where clustered into sub-clades using the Partitioning around Medoids algorithm (Kaufman and Rousseeuw, 1990) implemented in the cluster R package. The optimal number of clusters was determined using the prediction strength metric (Tibshirani and Walther, 2005) which supported two sub-clades for the *P. copri* Clades A, B and C.

Iceman Samples and Mexican Coprolite Material

In this study we metagenomically analyzed archaeological gut contents for the presence of *P. copri*. The analyzed material includes gut content and lung tissue (negative control) of the Iceman, a European Copper Age ice mummy (Figure 5A). The Iceman, commonly referred to as “Ötzi”, is one of the oldest human mummies discovered. His body was preserved for more than 5,300 years in an Italian Alpine glacier before he was discovered by two German mountaineers at an altitude of 3,210 m above sea level in September 1991. The mummy is now conserved at the Archaeological Museum in Bolzano, Italy, together with an array of accompanying artefacts (www.iceman.it). The Iceman was naturally mummified by freeze-drying (Lynnerup, 2007). Therefore, his body tissues and intestines still contain well preserved ancient biomolecules (DNA, proteins, lipids) that allowed e.g. the reconstruction of the Iceman’s genome (Keller et al., 2012), the genomic analysis of the stomach pathogen *Helicobacter pylori* (Maixner et al., 2016), and the molecular reconstruction of the Iceman’s last meal (Maixner et al., 2018). In addition, we subjected three ancient coprolite samples from a Mexican cave to metagenomics analysis (Figure 5A). The archaeological site “La Cueva de los Muertos Chiquitos” in the northern Durango region of el Zape, Mexico, was excavated by Brooks and colleagues in the early 1960s (Brooks et al., 1962). The sub-humid climate in this natural cave at an altitude of approx. 1,800 m above sea level provided favorable conditions for the preservation of various ancient remains including human skeletons, botanical artefacts, quids and coprolites. The site was dated by previous radiocarbon dating of a single wood sample from one of the oldest levels (square B4, level 24-28) to AD 600 (1300 ± 100 BP) (Brooks et al., 1962). The ancient remains have been previously subjected to botanical (Brooks et al., 1962), dietary (Hammerl et al., 2015; Meade, 1994), parasitological (Camacho et al., 2018; Jiménez et al., 2012; Morrow and Reinhard, 2016), and molecular analysis (Tito et al., 2008, 2012). All three coprolite samples used in this study were discovered in square B4 in two different levels (Table S4). The samples were stored in the Pathoecology Laboratory in the School of Natural Resources at the University of Nebraska-Lincoln in Lincoln, Nebraska. We obtained radiocarbon dates at the Curt-Engelholm-Centre for Archaeometry, Mannheim, Germany for coprolite sample 2180 from level 16-20 (AD 673 to 768, 1,284 ± 16 BP) that confirms the previous direct dating of the pre-Columbian archaeological site (Table S4).

The molecular analysis of the Iceman samples and of the ancient human coprolites was conducted at the ancient DNA laboratory of the EURAC Institute for Mummy Studies in Bolzano, Italy. Sample preparation and DNA extraction was performed in a dedicated pre-PCR area following the strict procedures required for studies of ancient DNA: use of protective clothing, UV-light exposure of the equipment and bleach sterilisation of surfaces, use of PCR workstations and filtered pipette tips. DNA was extracted from the archaeological specimen using a chloroform-based DNA extraction method according to the protocol of (Tang et al., 2008). Libraries for the sequencing runs were generated with a modified protocol for Illumina multiplex sequencing (Kircher et al., 2012; Meyer and Kircher, 2010). Libraries of the Mexican coprolite samples were sequenced on Illumina HiSeq2500 platforms using 101–base pair paired-end sequencing kits. The Iceman samples were sequenced on an Illumina HiSeqX platform using the 150–base pair paired-end sequencing kit.

Paired Illumina reads were quality-checked and processed (adapter removal and read merging) as previously described in (Maixner et al., 2018). Reads were mapped using Bowtie2 (Langmead and Salzberg, 2012) to the human genome (build Hg19, default mapping parameters) (Rosenbloom et al., 2015), the human mtDNA reference genome (rCRS, mapping parameter –very-sensitive-local) (Andrews et al., 1999), and selected *P. copri* genomes from the four clades. For details to the mapping results please refer to Table S4. To deduplicate the mapped reads we used the DeDup tool (https://github.com/apeltzer/DeDup). The minimum mapping and base quality were both 30. The resulting bam files were used to check for characteristic aDNA nucleotide misincorporation frequency patterns using mapDamage2 (Jónsson et al., 2013). Both human and bacterial reads display low but already increased frequencies of C to T substitutions close to the fragment ends characteristic of ancient DNA (Orlando et al., 2015) (Figures S5A and S5B). Reads of the Iceman lung tissue metagenome that mapped to one single marker of the 2,448 *P. copri* complex specific markers display no DNA damage. The sex of the mapped human reads was assigned using a Maximum likelihood method, based on the karyotype frequency of X and Y chromosomal reads (Skoglund et al., 2013) (Table S4). Estimation of human contamination rates using Schmutzi (Renaud et al., 2015) and ANGSD (Korneliussen et al., 2014) was in most samples not possible due to low damage pattern rates in the mitochondrial reads and due to the low coverage of the X chromosome (sample 2180), respectively.

The Iceman samples with sufficient X-chromosome coverage show low contamination in the autosomal DNA when using ANGSD (Table S4). Analysis of the human mitochondrial and autosomal variants provided further evidence for the sample origin and authenticity of the data. Variants in the mitochondrial genome were called using SAMtools mpileup and bcftoools (Li et al., 2009) with stringent filtering options (quality>30). Visual inspection of the called variants identified only less than 1% low-frequency variants that could be indicative for contamination. The haplogroup was identified by submitting the variant calling file to the HaploGrep website (Weissensteiner et al., 2016) (Table S4). The human mitochondrial genomes in both Iceman samples carry the same variants as reported in previous Iceman genomic studies and belong to the K1f haplotype (Ermini et al., 2008; Keller et al., 2012). Importantly, in the Mexican coprolite samples 2179 and 2180 both detected mitochondrial haplogroups (C1b and B2) belong to the four main pan-American mtDNA lineages (Achilli et al., 2008; Bodner et al., 2012; Perego et al., 2010; Tamm et al., 2007). Furthermore, both haplogroups have been detected in previous studies in ancient human remains from Meso- and South America (Fehren-Schmitz et al., 2015; Gómez-Carballa et al., 2015; Llamas et al., 2016; Posth et al., 2018; Tackney et al., 2015) and haplogroup C1b has still nowadays its highest frequency in Peru and Mexico (Gómez-Carballa et al., 2015). We extended our analysis to the human autosomal data and called pseudodiploid genotypes using SAMtools mpileup (Li et al., 2009) and PileupCaller (https://github.com/stschiff/sequenceTools/tree/master/src-pileupCaller) for the Mexican specimen with the highest endogenous human content (2179, 2180) at loci that over-lapped with the Affymetrix Human Origins SNP array data (Patterson et al., 2012) and merged them to a modern European, Asian and Native American subset (n=2068) (Lazaridis et al., 2016). Principal Component Analysis (PCA) (Patterson et al., 2006; Price et al., 2006) on the resulting SNP dataset show that the human DNA form the two coprolite samples has the greatest genetic affinity with modern Native Americans (Figure S5D). This result highly supports the haplogroup assignment of the uniparental marker and genetically allocates the specimens to the American continent.

P. copri Genome Reconstruction from Ancient Gut Metagenomes and Molecular Dating

To reconstruct ancient *P. copri* genomes, we utilized our in-house scripts (https://bitbucket.org/CibioCM/cmseq) to build 4 ancient *P. copri* genome ‘‘scaffolds’’, extracting consensus sites of aligned reads of sample 2179 and 2180 (the two samples with all four copri clades detected, Figure 5B) to representative genomes, one for each of the four clades for *P. corpi* complex. Sites covered by ancient reads were filled with gaps if one of following quality criteria was violated: (1) mapping quality is less than 30, (2) coverage is less than 5-fold, (3) the length of aligned read is less than 50nt, (4) minimum identity for the read is less than 97%, (5) minimum dominant allele frequency is less than 80%. Phylogeny of each core gene of a total of 540 was analyzed separately using BEAST (version 2.5.1) (Bouckaert et al., 2014). Core genes (n=210) supporting monophyly of the 4 *P. copri* clades (thus are unlikely to have been subject of horizontal gene transfer) were kept for searching for orthologs in ancient *P. copri* and their modern counterparts. We searched for these orthologs by aligning selected core genes against 4 ‘‘scaffolds’’ representative of the clades using Blastn (Altschul et al., 1990) with parameter -word_size of 9. Mapping hits with either length less than 30 bp or e-value over 1e-10 were excluded. We kept orthologs shared by all 72 modern *P. copri* genomes and at least 1 ancient *P. copri* genome ‘‘scaffold’’, and subsequently applied multiple sequence alignment using MAFFT (Katoh and Standley, 2013), with parameter –maxiterate of 1000 and –globalpair, to each of orthologs. Single-ortholog alignments were manually curated excluding mis-aligned sites (we consider continuous variant nucleotides observed in the alignment as artificially mis-aligned sites) and were then merged into one concatenation alignment.

Out of eight ancient strains, we chose only the one with the best overall coverage which was the Clade A strain from sample 2180 (Figure S5E). This sample was accurately radiocarbon dated (AD 673 to 768, 1284 ± 16 BP). The alignment composed of the selected ancient *P. copri* starin and 72 modern strains, which was further processed to automatically remove gappy columns using trimAl (Capella-Gutiérrez et al., 2009). The final alignment included 214,399 nucleotide positions. BEAST (version 2.5.1) (Bouckaert et al., 2014) was used to infer divergence times of *P. copri* clades, using a GTR model of nucleotide substitution (with 4 gamma categories). To choose the best clock and demographic models we performed a model selection comparing coalescent constant, coalescent exponential, coalescent bayesian skyline, and coalescent extended bayesian skyline models (for the demographic priors) and strict and relaxed lognormal (for the clock prior). Model selection (Table S4) was performed by comparing AICM from BEAST analyses with 100,000,000 Markov Chain Monte Carlo (MCMC) states for each model and sampling every 10,000 states. Convergence of posteriors was assessed by visualising log files with Tracer (version 1.7) (Rambaut et al., 2018). The most fitting combination of models was a coalescent constant population, with strict clock: this analysis was run longer for 204,000,000 iterations and effective sample size (ESS) of all parameters was over 200. To confirm age estimates of each clade, the same molecular clocking analysis was performed independently on each *P. copri* clade using the corresponding ancient strain.

QUANTIFICATION AND STATISTICAL ANALYSIS

All statistical significance was performed using the Welch’s t-test, Fisher’s exact test or Mann-Whitney U test and where applicable corrected for multiple hypothesis testing using the Bonferroni method. All computational and statistical analyses were performed using open-source software and referenced in the Key Resources Table and methods and described in the main text and STAR Methods.

DATA AND CODE AVAILABILITY

The 15 isolate *P. copri* genomes (and the extended set of 83 isolate, see above) and all metegenomically assembled metagenomes (MAGS) are available here: http://segatalab.cibio.unitn.it/data/Pcopri_Tett_et_al.html and metadata given in Tables S1 and S2. The full *P. copri* phylogeny of 1023 genomes is available at http://segatalab.cibio.unitn.it/data/Pcopri_Tett_et_al.html. Metadata for the three sequenced non-Westernized dataset is given in Table S5 and is also available as part of the *curatedMetagenomicData* package (Pasolli et al., 2017). The metagenomic reads for these datasets are available under NCBI-SRA BioProject ids; NCBI: PRJNA529124 (Ghana), NCBI: PRJNA529400 (Tanzania), NCBI: PRJNA504891 (Ethiopia). The Data for the ancient metagenomic samples are available under accession NCBI: PRJEB31971.

Cell Host & Microbe, Volume *26*

Supplemental Information

The *Prevotella copri* Complex Comprises Four Distinct Clades Underrepresented in Westernized Populations

Adrian Tett, Kun D. Huang, Francesco Asnicar, Hannah Fehlner-Peach, Edoardo Pasolli, Nicolai Karcher, Federica Armanini, Paolo Manghi, Kevin Bonham, Moreno Zolfo, Francesca De Filippis, Cara Magnabosco, Richard Bonneau, John Lusingu, John Amuasi, Karl Reinhard, Thomas Rattei, Fredrik Boulund, Lars Engstrand, Albert Zink, Maria Carmen Collado, Dan R. Littman, Daniel Eibach, Danilo Ercolini, Omar Rota-Stabelli, Curtis Huttenhower, Frank Maixner, and Nicola Segata

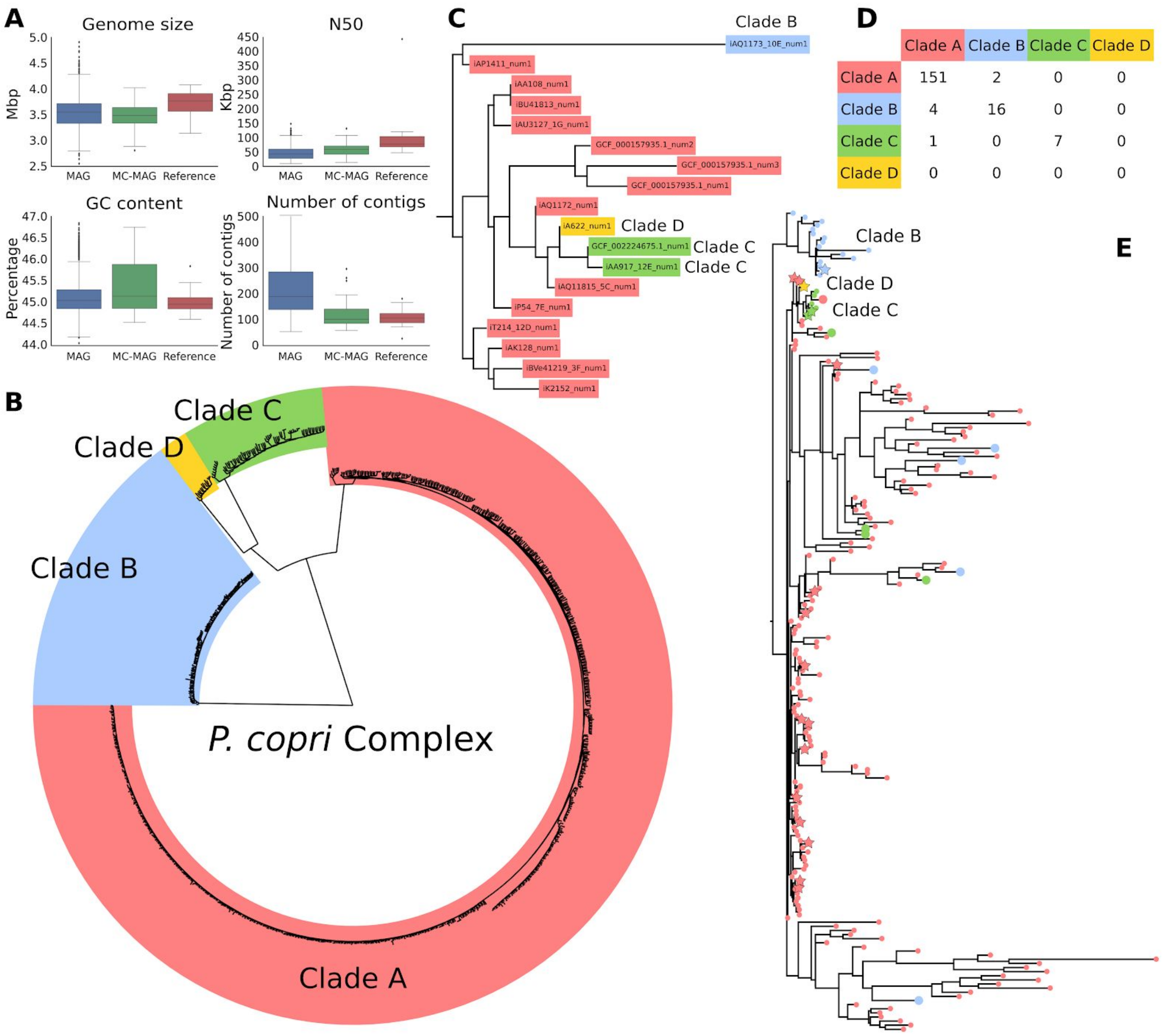

Supplementary Figure S1. ***P. copri*** **genome statistics and phylogenetic relatedness, related to Figure 1. A)** Comparison of genome statistics (Genome size, N50, GC% and number of contigs) for *P. copri* isolate sequences (references = 17), for manually curated metagenome assembled genomes (MC-MAG = 55) and automatically metagenome assembled genomes (MAGs = 951). **B)** Phylogenetic representation of all 1023 *P. copri* genomes based on a set of 210 *P. copri* core genes (see **Methods**). **C-E**. Relatedness of the 16S rRNA gene sequences of the four *P. copri* clades reveals weak resolving power in discriminating the four clades. **C**, Phylogeny of all 16S rRNA gene sequences (>1000bp) recovered from all 17 isolate genomes using Barnap (https://github.com/tseemann/barrnap). **D**, Confusion matrix of all recovered 16S sequences (>1000bp) from all MAGs and their clade membership assigned based on the 16S rRNA gene sequence of the closest isolate genome (Blastn, >500bp alignment, identity > 85%) compared to their clade membership based on whole genome phylogenetic placement (panel **B**). **E**, 16S rRNA phylogenetic representation of all isolate genomes and MAGs. All alignments were produced using MAFFT (Katoh and Standley, 2013) and the following parameters: mafft --globalpair --maxiterate 1000 and visualised using FastTree with default parameters (Price et al., 2010).

A

# sample	Prevalence (Fisher) Any	Clade A	Clade B	Clade C	Clade D	Abundance (Mann-Whitney) Any	Clade A	Clade B	Clade C	Clade D	Dataset	Condition
66	27.3	21.2	7.6	12.1	3.0	6.7	4.1	0.4	7.6	0.6	ZellerG_2014	Control
42	35.7	33.3	11.9	9.5	4.8	11.6	8.8	1.5	4.2	13.5		Adenoma
91	26.4	20.9	11.0	7.7	4.4	6.6	5.4	2.4	0.9	6.4		CRC
133	29.3	24.8	11.3	8.3	4.5	8.6	6.8	2.1	2.1	8.8		Adenoma/CRC
61	8.2	3.3	1.6	3.3	1.6	1.0	0.5	1.5	0.8	1.1	FengQ_2015	Control
46	**39.1 ***	**39.1 ***	4.3	**17.4 ***	0.0	**7.0 ***	**6.4 ***	0.9	**1.1 ***	0.0		CRC
47	17.0	12.8	4.3	6.4	0.0	2.0	1.7	0.5	1.6	0.0		Adenoma
93	**28.0 ***	**25.8 ***	4.3	11.8	0.0	**5.5 ***	**5.3 ***	0.7	1.2	0.0		CRC/adenoma
52	23.1	23.1	1.9	0.0	0.0	11.7	11.7	0.2	0.0	0.0	VogtmannE_2016	Control
52	19.2	19.2	0.0	0.0	0.0	12.6	12.6	0.0	0.0	0.0		CRC
53	28.3	24.5	1.9	7.5	0.0	10.2	9.8	0.3	6.3	0.0	YuJ_2015	Control
75	30.7	28.0	5.3	4.0	0.0	10.0	10.2	2.2	2.7	0.0		CRC
174	29.9	25.9	4.0	8.0	1.7	28.6	26.4	10.2	15.1	5.2	QinJ_2012	Control
170	28.2	25.3	6.5	10.0	1.8	17.2	13.7	7.3	8.1	6.9		T2D
43	14.0	11.6	0.0	7.0	0.0	2.1	1.6	0.0	1.5	0.0	KarlssonFH_2013	Control
102	16.7	14.7	2.0	2.9	2.0	4.4	3.7	0.5	4.0	2.8		IGT/T2D
49	10.2	6.1	2.0	4.1	0.0	2.6	1.5	0.2	4.1	0.0		IGT
53	22.6	22.6	1.9	1.9	3.8	5.1	4.3	0.8	4.0	2.8		T2D
41	39.0	39.0	7.3	4.9	0.0	31.2	29.7	2.5	8.5	0.0	LiJ_2017	Control
99	48.5	47.5	11.1	16.2	2.0	37.3	31.6	4.2	13.8	18.6		Hypertension
56	53.6	48.2	3.6	**19.6 ***	1.8	41.4	39.0	11.4	**14.9 ***	2.1		Pre-hypertension
155	50.3	47.7	8.4	**17.4 ***	1.9	38.9	34.3	5.3	**14.2 ***	13.1		Pre-hypertension/hypertension
71	25.4	25.4	1.4	2.8	0.0	8.6	8.1	0.4	4.6	0.0	NielsenHB_2014	Control
148	33.8	29.7	**11.5 ***	3.4	**6.1 ***	11.5	10.1	**2.8 ***	8.9	**4.5 ***		IBD
53	26.4	24.5	1.9	11.3	3.8	15.1	12.5	1.1	6.7	3.4	HeQ_2017	Control
63	12.7	11.1	4.8	3.2	0.0	45.9	31.6	17.6	46.6	0.0		CD
114	52.6	48.2	1.8	13.2	1.8	9.6	9.1	4.8	3.9	2.1	QinN_2014	Control
123	58.5	57.7	2.4	16.3	0.8	11.7	9.7	1.5	6.8	14.5		Cirrhosis
36	11.1	8.3	5.6	2.8	0.0	25.4	26.2	10.0	3.0	0.0	RaymondF_2016	Control
36	19.4	13.9	11.1	5.6	0.0	16.0	17.6	4.6	2.8	0.0		Cephalosporins

B

#Samples	Clade A Sub-1	Clade A Sub-2	Clade B Sub-1	Clade B Sub-2	Clade C Sub-1	Clade C Sub-2	Dataset	Condition
93	5.38	1.08	0.00	1.08	2.15	0.00	FengQ_2015	CRC/ADENOMA
61	0.00	0.00	0.00	1.64	0.00	0.00		Control
133	4.51	6.02	0.00	0.75	0.00	0.00	ZellerG_2014	CRC/ADENOMA
66	6.06	1.52	0.00	0.00	1.52	0.00		Control
52	3.85	13.46	0.00	0.00	0.00	0.00	VogtmannE_2016	CRC
52	0.00	21.15	0.00	0.00	0.00	0.00		Control
75	10.67	6.67	0.00	1.33	0.00	0.00	YuJ_2015	CRC
53	13.21	3.77	0.00	0.00	0.00	3.77		Control
170	10.59	2.94	0.00	1.76	2.35	0.00	QinJ_2012	T2D
174	14.94	2.87	0.00	1.72	1.72	0.57		Control
102	2.94	4.90	0.00	0.00	0.00	0.00	KarlssonFH_2013	IGT/T2D
43	2.33	0.00	0.00	0.00	0.00	2.33		Control
155	14.19	12.26	0.00	1.29	0.65	2.58	LiJ_2017	Pre-hypertension/hypertension
41	12.20	12.20	0.00	0.00	0.00	0.00		Control
148	6.76	7.43	0.00	0.68	0.00	0.00	NielsenHB_2014	IBD
71	8.45	8.45	0.00	0.00	0.00	0.00		Control
63	6.35	0.00	0.00	1.59	1.59	0.00	HeQ_2017	CD
53	9.43	0.00	0.00	0.00	0.00	3.77		Control
123	16.26	2.44	0.00	0.00	0.00	1.63	QinN_2014	Cirrhosis
114	11.40	3.51	0.00	1.75	0.88	0.88		Control
36	0.00	5.56	0.00	8.33	0.00	0.00	RaymondF_2016	Cephalosporins
36	0.00	2.78	0.00	2.78	0.00	0.00		Control

Supplementary Figure S2. The *P. copri* complex with respect to metagenomically investigated human diseases. Related to Figure 2. A, Prevalence and abundance of the *P. copri* complex in publicly available datasets for which there are case and control samples. * indicates p <0.05 (Fisher exact test (prevalence) or Mann-Whitney U test (abundance)) **B**, There is no significant association of *P. copri* sub-clades and disease (Fisher exact test), see **Methods** for inference of sub-clades.

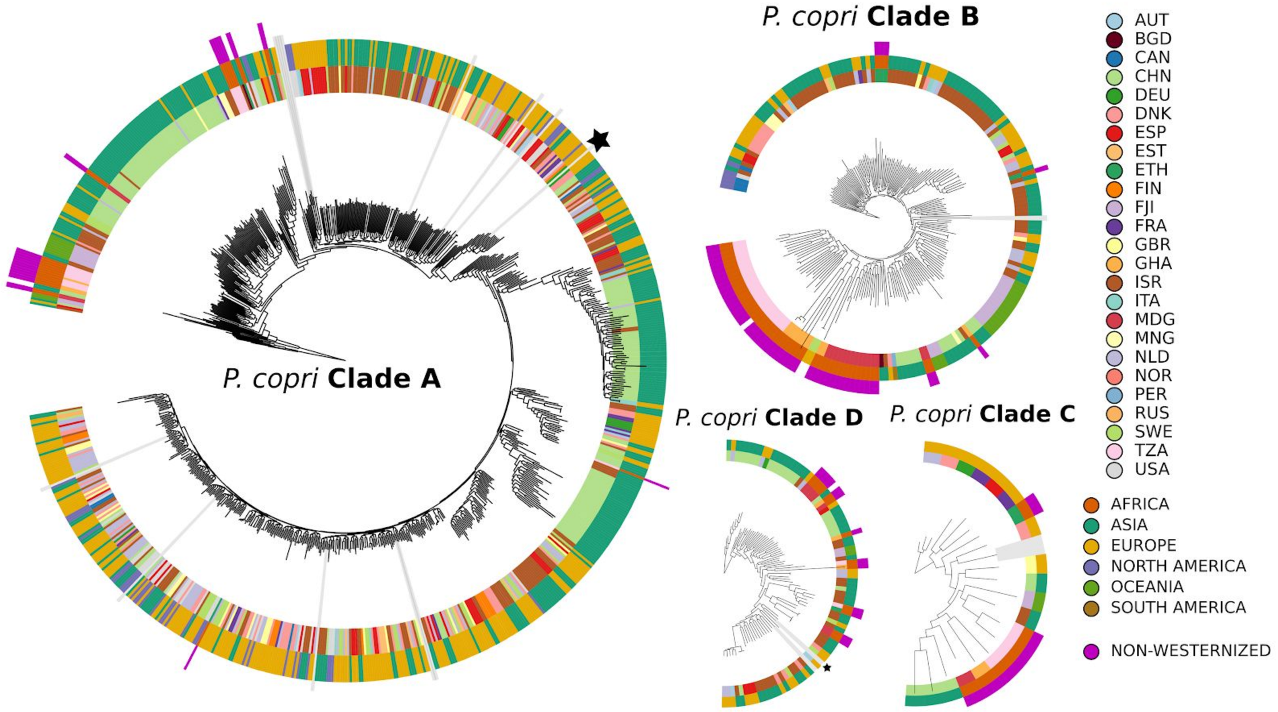

Supplementary Figure S3. Phylogeny of all 1121 *P. copri* genomes reconstructed in this study. Related to Figure 3. Outermost ring indicates if the genome was reconstructed from a non-Westernized sample (which includes the 98 genomes from our recently sequenced non-Westernized datasets), middle ring the continent and inner ring the country of origin. Publicly available *P. copri* references are indicated by black stars and our isolate genomes by radial gray bars.

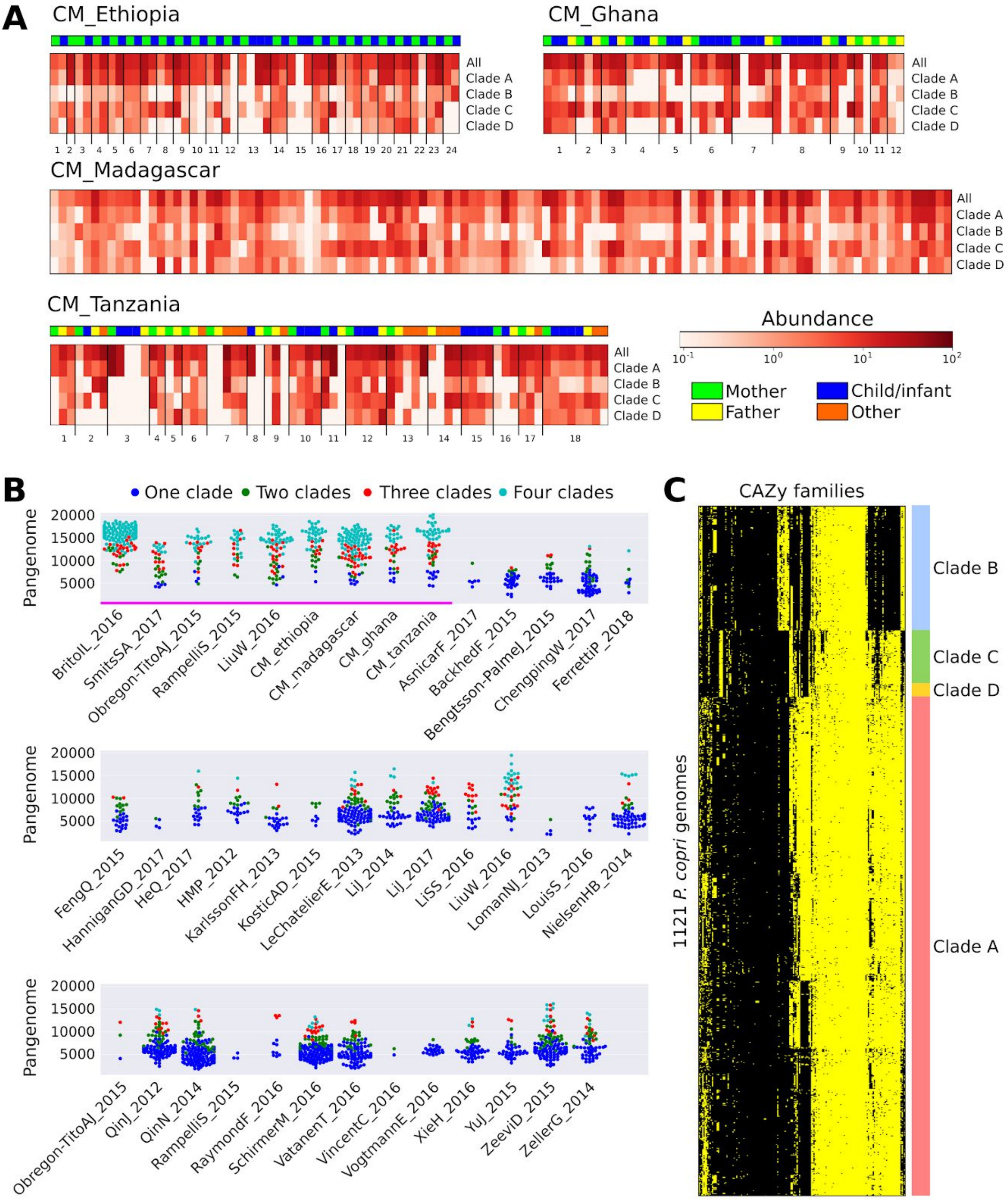

Supplementary Figure 4. **Abundance of *P. copri* in the recently sequenced non-Westernized datasets and functional diversity of the *P. copri* complex. Related to Figures 3 and 4. A**, Co-presence and abundance of the *P. copri* complex in our recently sequenced Non-westernized datasets. For datasets from Ethiopia, Ghana and Tanzania numbers refers to family membership. **B**, The within sample *P. copri* complex pangenome for datasets considered in this study. Non-Westernized datasets are underlined in magenta. **C**, Presence/absence heatmap of CAZy families in each of the 1121 *P. copri* genomes (yellow present, black absent)

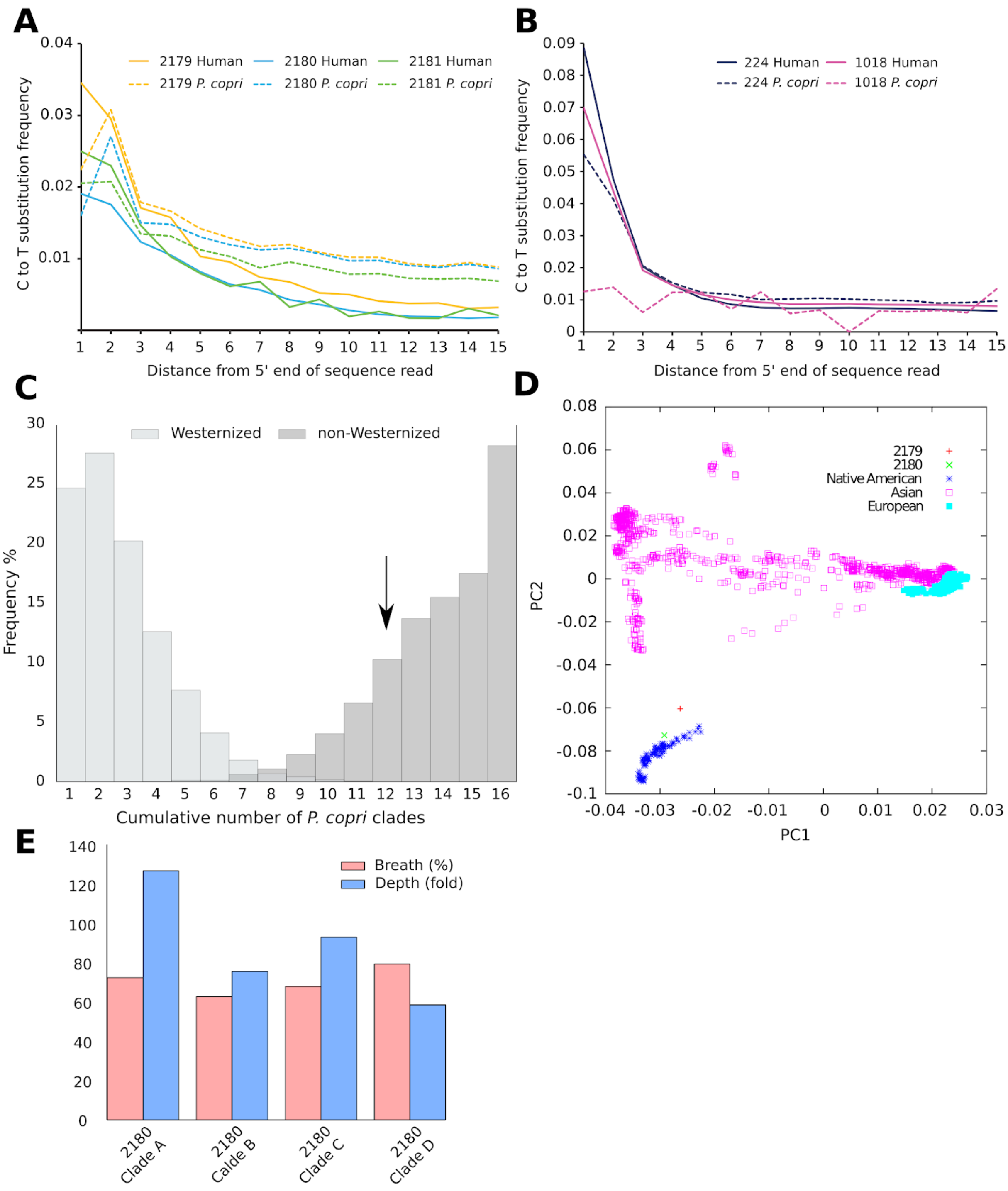

Supplementary Figure S5. **Analysis of the ancient ice-mummy and pre-Columbian amerind metagenomic samples. Related to Figure 5 and STAR methods.** Ancient DNA damage profiles. Cytosine to thymine substitution frequencies in the 5´ end of the human and *P. copri* (dashed lines) sequence reads detected in the Mexican coprolite material **(A)** and in the Iceman samples **(B)**. **C**, Co-presence of *P. copri* clades in ancient individuals is similar to contemporary non-Westernized individuals. Random subsampling of four individuals from either non-Westernized or Westernized populations and the cumulative number of *P. copri* clades observed (min 0, max 16). Subsampling repeated 10,000 times for each population. Black arrow indicates the number observed in the four ancient samples. **D**, PCA plot of two Mexican coprolite samples and selected modern European, Asian and Native American. Genome-wide ancient data was projected against a selected subset of the Affymetrix Human Origins populations. **E**, Depth and breadth of metagenomic reads from

sample 2180 mapped against four *P. copri* isolate genomes representing the four clades in the *P. copri* complex.
